# Metabolic interactions in the brain: the crucial roles of neurons, astrocytes, and microglia in health and disease

**DOI:** 10.3389/fnins.2026.1731771

**Published:** 2026-02-03

**Authors:** Yonghui Pang, Jiachen Yang, Jingjing Liu, Zhengyi Xie, Jin Wang

**Affiliations:** 1 The First Clinical Medical School, Guilin Medical University, Guilin, China; 2 Department of Gastrointestinal Surgery, The Second Affiliated Hospital of Guangxi University of Science and Technology, Liuzhou, China; 3 Department of Medicine, Guangxi University of Science and Technology, Liuzhou, China

**Keywords:** astrocytes, disease, homeostasis, metabolism, microglia, neurons

## Abstract

This review provides an in-depth exploration of the intricate energy metabolism pathways within the brain, with a particular focus on the dynamic interplay between neurons, astrocytes, and microglia. Neurons, with their high energy demands, primarily rely on oxidative phosphorylation and the tricarboxylic acid (TCA) cycle to sustain synaptic activity and neurotransmitter synthesis. In contrast, astrocytes predominantly engage in glycolysis, producing lactate and glutathione, which are essential for supporting neuronal function and protecting against oxidative stress. Additionally, microglia, the brain’s resident immune cells, exhibit a metabolic flexibility that allows them to shift between oxidative phosphorylation and glycolysis, depending on their activation state, which significantly influences neuroinflammation and synaptic plasticity. The review highlights the critical role of astrocyte-neuron metabolic coupling, particularly through the lactate shuttle and glutathione metabolism, in maintaining neuronal homeostasis and facilitating synaptic function. It also delves into the metabolic underpinnings of neurodegenerative diseases such as Alzheimer’s, Parkinson’s, and Amyotrophic Lateral Sclerosis, illustrating how disruptions in brain energy metabolism contribute to disease progression. By synthesizing recent findings, this review not only underscores the centrality of brain energy metabolism in both normal and pathological conditions but also identifies potential therapeutic targets aimed at modulating these metabolic pathways to mitigate the effects of neurodegenerative disorders. This comprehensive analysis offers valuable insights that could propel further research and innovation in the field of neurology, making it essential reading for experts interested in the molecular mechanisms underlying brain function and disease.

## 1 Introduction

The brain, as the largest and most intricate structure in the central nervous system, functions as the core hub for information processing in humans (Hernández-Peón and Sterman, 1966). It is responsible for a range of advanced functions, including the generation and maintenance of consciousness (Mashour et al., 2020), as well as the formation and regulation of emotions (Lindquist et al., 2012). Furthermore, the brain provides the essential material foundation for learning (Meyniel and Dehaene, 2017), both short- and long-term memory (Hanslmayr et al., 2019), comprehension, and language (Tagarelli et al., 2019). The efficient processing and transmission of information within the brain are reliant on synaptic transmissions facilitated by action potentials (Bykhovskaia and Vasin, 2017; Schmidt and Knösche, 2019). To sustain these sophisticated and demanding functions, the brain depends on an intricate network of interconnected neural circuits (Williams, 2016), which encompass a variety of energy-intensive physiological processes. These processes include the maintenance of ionic electrochemical gradients (Farsi et al., 2017), the regulation of ion channels (Becchetti et al., 2019), and the management of neurotransmitter release, uptake, and recycling (Armbruster et al., 2020; Ge et al., 2020). As a result, the brain consumes a substantial amount of energy to sustain its physiological activities. Despite constituting only 2% of total body weight, the brain accounts for approximately 20% of the body’s total oxygen and glucose consumption, underscoring the critical importance of brain energy metabolism as a focal point of scientific research (Bélanger et al., 2011).

In the adult brain, glucose, primarily derived from the circulatory system, serves as the essential energy substrate, fulfilling the brain’s diverse metabolic needs through various complex pathways (Dienel, 2019). These pathways include: (1) the direct production of lactic acid, adenosine triphosphate (ATP), and NAD + via glycolysis, independent of mitochondrial involvement; (2) the conversion of glucose to pyruvate through glycolysis, followed by its entry into the mitochondria via pyruvate dehydrogenase, leading to substantial ATP production and carbon dioxide release through the tricarboxylic acid (TCA) cycle and oxidative phosphorylation; (3) the synthesis of significant amounts of reduced nicotinamide adenine dinucleotide phosphate (NADPH) via the pentose phosphate pathway, which acts as a reducing agent against oxidative stress and generates ribose 5-phosphate for purine nucleotide biosynthesis; and (4) the conversion of glucose to glycogen via glucose-6-phosphate (Glc-6-P), providing an energy reserve for periods of glucose deprivation and certain pathophysiological conditions (Bak et al., 2018; Duran et al., 2019).

Moreover, the by-products of glycolysis contribute to the synthesis of essential compounds necessary for cellular structure and function, including amino acids (e.g., serine, glycine, alanine, and glutamine), neurotransmitters, neuromodulators (e.g., glutamate, GABA, glycine, and acetylcholine), glycolipids, and glycoproteins (Dienel, 2019). The brain consists of various cell types, including neurons and glial cells (astrocytes, microglia, and oligodendrocytes), each playing a crucial role in maintaining brain function. These cells often work synergistically, despite having distinct energy metabolism profiles (Table 1) (Bélanger et al., 2011). Among them, neurons and astrocytes are closely linked through various metabolic interactions, including the lactate shuttle (Brooks, 2018) and glutathione (GSH) circulation (Dwivedi et al., 2020). Therefore, elucidating the differences and interconnections in the metabolism of various brain cell types is essential for understanding their respective roles in brain function.

**Table 1:** Metabolic characteristics of major brain cell types.

Cell type	Main metabolic pathway	Key enzyme/transporter protein	Energy substrate preference	Main metabolites
Neuron	Oxidative phosphorylation TCA cycle Limited glycolysis	MCT2 LDH1 low PFKFB3	Lactic acid Glucose 2DG-IR (glucose analog)	NADPH ATP Neurotransmitters
Astrocyte	High-glucose glycolysis Glycogen synthesis and decomposition Glutamic acid metabolism	PFKFB3(high) MCT1/4, GLT-1/GLAST LDH5,γ-GT Pyruvate dehydrogenase	Glucose (high uptake) Cys	Lactic acid Glutathione (GSH) fructose-2,6-bisphosphate
Microglia	Resting state: oxidative phosphorylation Activated state: glycolysis	GLUT HK2 AMPK-mTOR-HIF-1α axis	Glucose Glutamic acid SLC1A5 SLC38A1	Lactic acid Cytokines

Given the pivotal role of energy metabolism in neuronal function and survival, current research is increasingly focused on uncovering the nuanced regulatory mechanisms governing these metabolic pathways. Advances in single-cell transcriptomics and metabolomics are enabling researchers to explore metabolic heterogeneity at unprecedented resolutions. These techniques are expected to reveal how individual neurons and specific subpopulations within the brain adapt their energy metabolism under varying physiological and pathological conditions (Barros, 2013; Magistretti and Allaman, 2015).

Another emerging research trend involves the investigation of metabolic reprogramming in neurons during neurodegenerative diseases. For instance, alterations in glucose and lactate metabolism have been implicated in the early stages of Alzheimer’s and Parkinson’s diseases (Bolaños et al., 2010). Understanding these metabolic reprogramming processes—such as PDK4-mediated pyruvate dehydrogenase inhibition and PKM2/PKM1 isoform switching—could identify novel therapeutic targets by regulating key pathways like Anaphase-promoting complex/cyclosome and its coactivator Cdh1 (APC/C-Cdh1)-dependent PFKFB3 stabilization or pentose phosphate pathway activation. Such interventions may restore metabolic balance in neurons, potentially slowing disease progression by improving energy production and reducing oxidative stress (Ashrafi and Ryan, 2017).

Furthermore, the interplay between neuronal energy metabolism and synaptic plasticity is an area of growing interest. Evidence suggests that metabolic substrates, such as lactate, may play a signaling role in synaptic modulation and plasticity (Möller et al., 2021). Exploring these links could offer new insights into how metabolic processes influence cognitive functions, such as learning and memory (Yellen, 2018).

Finally, future research may delve into the potential of metabolic interventions as therapeutic strategies. For example, enhancing neuronal glucose uptake and utilization or modulating specific metabolic pathways could provide protective effects against metabolic stress and neurodegeneration (Riera and Dillin, 2015). With the advent of new technologies and a deeper understanding of neuronal metabolism, the field is poised for significant breakthroughs that could transform our approach to treating neurological disorders (Lee and Silva, 2009).

In conclusion, while the fundamental aspects of neuronal energy metabolism are well-documented, ongoing and future research will likely focus on the complex regulatory networks that enable neurons to maintain energy homeostasis, particularly under stress or disease conditions (Antal et al., 2025). Gaining a deeper understanding of these processes will be crucial for developing novel therapeutic approaches aimed at preserving neuronal function and preventing neurodegenerative diseases (Chih and Roberts, 2003).

Additionally, it is important to note that the field of brain energy metabolism encompasses lively debates regarding the predominant pathways supporting neuronal activation. While the astrocyte-neuron lactate shuttle (ANLS) hypothesis (Pellerin and Magistretti, 1994), which emphasizes lactate transfer and its subsequent oxidative metabolism, has gained substantial experimental support, alternative viewpoints exist. A significant body of work, often based on neuroimaging techniques such as positron emission tomography (PET) and blood-oxygen-level-dependent functional magnetic resonance imaging (BOLD-fMRI), has reported a disproportionate increase in glucose utilization relative to oxygen consumption during brain activation. This observation has been interpreted by some as evidence for predominant “non-oxidative glucose consumption” within activated tissue (Fox et al., 1988; Dienel, 2012, 2017, 2019). However, this interpretation is increasingly scrutinized. Critics argue that methodologies relying solely on hemodynamic signals or blood oxygen content may overlook oxygen stored within and consumed directly from the brain parenchyma, particularly in lipid-rich structures like myelin sheaths (Morelli and Scholkmann, 2023; Schurr, 2025). Recent studies providing mechanistic support for significant extravascular oxygen storage and its rapid utilization have revitalized the argument that oxidative metabolism, supported by substrates like lactate, is central to meeting activity-dependent energy demands (Schurr, 2025). Recent studies providing mechanistic support for significant extravascular oxygen storage and its rapid utilization have revitalized the argument that oxidative metabolism, supported by substrates like lactate, is central to meeting activity-dependent energy demands (Morelli and Scholkmann, 2023; Schurr, 2025). This review will present the evidence for metabolic coupling as framed by the ANLS hypothesis, while acknowledging and examining these ongoing methodological and interpretive debates where relevant.

## 2 Energy metabolism in neurons

### 2.1 Core characteristics and substrate preference of neuronal metabolism

Neurons, in comparison to glial cells, maintain a higher energy level due to their continuous need to generate action potentials, synaptic potentials, and neurotransmitters. Similar to glial cells, neurons are capable of fully oxidizing glucose and lactate. However, neuronal energy metabolism displays distinct characteristics, especially when responding to increased energy demand (see Table 1 for an overview).

A study employing high-resolution immunocytochemistry at both light and electron microscopic levels in rodents has demonstrated that neurons efficiently uptake lactate via monocarboxylate transporter 2 (MCT2), a high-affinity transporter localized at postsynaptic densities to facilitate lactate import from astrocyte-released sources (Bergersen, 2007) and subsequently convert it to pyruvate via lactate dehydrogenase isotype 1. When both glucose and lactate are available, neurons preferentially utilize lactate for energy production, with lactate contributing up to 75% to neuronal oxidative metabolism under physiological concentration conditions (Bouzier-Sore et al., 2006). Although neurons possess a full complement of glycolytic enzymes, their expression of 6-phosphofructo-2-kinase (PFK2) is extremely low, especially the Pfkfb3 isoform (Almeida et al., 2004). This enzyme catalyzes the production of fructose-2,6-bisphosphate, an activator of phosphofructokinase-1 (PFK), which, in turn, reduces the efficiency of glycolysis in neurons.

### 2.2 Expression and regulatory mechanisms of key molecules in neuronal metabolism

While Pfkfb3 expression is comparable between neurons and astrocytes, there is a significant difference in the degradation process within neurons. Specifically, Pfkfb3 undergoes rapid degradation through the ubiquitin-proteasome pathway in neurons, resulting in lower levels of Pfkfb3 compared to astrocytes (Riera et al., 2003). This distinction becomes particularly critical under conditions of mitochondrial dysfunction. In such scenarios, neurons are prone to spontaneous apoptosis due to insufficient ATP production, whereas astrocytes demonstrate greater resilience. Astrocytes can upregulate rapid glycolysis mediated by PFKFB, thereby enhancing energy production and mitigating mitochondrial dysfunction.

Beyond ATP production, neurons also depend on NADPH for antioxidant functions. As a key product of the pentose phosphate pathway, NADPH strengthens cellular antioxidant defenses by driving the regeneration of reduced glutathione (Bolaños et al., 2010). Additionally, lactate can be converted to pyruvate intra-mitochondrially via mitochondrial lactate dehydrogenase (mLDH), rather than by the cytosolic isoform (cLDH), a reaction that initiates entry into the tricarboxylic acid (TCA) cycle and oxidative phosphorylation.

However, overexpression of Pfkfb3 activates glycolysis in neurons through the APC/C-Cdh1 pathway, which inhibits the pentose phosphate pathway (PPP), reduces the oxidation of glucose-6-phosphate (G6P), decreases the production of reduced glutathione, and finally leads to oxidative stress and apoptosis (Herrero-Mendez et al., 2009). Meanwhile, the enhancement of glycolysis inhibits the pentose phosphate pathway (PPP), resulting in a decrease in the oxidation rate of G6P and a reduction in the production of reduced glutathione (Tietze, 1969). Notably, this inhibitory effect has no impact on the apoptosis of astrocytes (García-Nogales et al., 2003). These observations suggest that neurons have a limited capacity to sustain high rates of glycolysis, warranting further investigation into whether neurons can enhance glycolytic rates under specific conditions.

Moreover, studies have shown that neurons can meet their energy demands by increasing the efficiency of glucose uptake and metabolism in response to stimulation. For example, using ^13^C-labeled 2-fluoro-2-deoxy-D-glucose (FDG) combined with nuclear magnetic resonance, a study found that glucose phosphorylation in nerve terminals significantly increases during acute neuronal activity (such as seizures), confirming the activity-dependent glucose uptake and glycolytic capacity of neurons. Studies observed through two-photon imaging that neurons in awake mice preferentially uptake the glucose analog 2-deoxyglucose-IR (2DG-IR) during sensory stimulation, with higher hexokinase expression than astrocytes, indicating that neurons have an intrinsic ability to metabolize glucose through enhanced glycolysis (Tang, 2018; Díaz-García and Yellen, 2019).

### 2.3 Dynamic regulation of neuronal metabolism and advances in research technologies

As our understanding of neuronal energy metabolism deepens, current research increasingly focuses on the dynamic regulation of metabolic pathways within neurons under diverse physiological and pathological conditions. Advances in single-cell transcriptomics, metabolomics, and live-cell imaging—such as the genetically encoded nicotinamide adenine dinucleotide (NADH) sensor Peredox—enable researchers to dissect the metabolic profiles of individual neurons and glia with unprecedented precision. For example, single-cell sequencing has revealed that neurons upregulate glucose transporter GLUT3 and hexokinase 2 gene expression under oxidative stress, while live-cell imaging directly visualizes mitochondrial reorganization and glycolytic enzyme relocalization during surges in energy demand. These techniques are unraveling how neurons finely tune the balance between glycolysis and oxidative phosphorylation to cope with challenges like oxidative stress or mitochondrial dysfunction (Barros, 2013).

### 2.4 Association between neuronal metabolic reprogramming and neurodegenerative diseases

A key area of interest is the interplay between neuronal energy metabolism and neurodegenerative diseases. Recent studies have revealed that neuronal metabolic reprogramming, characterized by PDK4-mediated inhibition of pyruvate dehydrogenase and altered expression of PKM2/PKM1 isoforms, contributes to early-stage Alzheimer’s and Parkinson’s diseases. This reprogramming leads to a metabolic shift where a greater proportion of glucose is diverted through glycolysis to produce lactate, instead of being fully oxidized in mitochondria. This results in cellular energy deficits and increased oxidative stress (Magistretti and Allaman, 2015). Understanding these processes may uncover novel therapeutic targets aimed at restoring metabolic balance in affected neurons, potentially slowing or even reversing disease progression (Bolaños et al., 2010).

### 2.5 Metabolic coupling mechanisms between neurons and astrocytes

Another emerging trend is the investigation of metabolic coupling between neurons and astrocytes (Ashrafi and Ryan, 2017). *In vivo* studies using two-photon fluorescence lifetime imaging and genetically encoded sensors (e.g., the laconic lactate biosensor) have confirmed that astrocytes generate lactate via glycolysis during neuronal activation, which is transported to neurons through monocarboxylate transporter MCT2. This process not only supplies an oxidative energy substrate but also enhances synaptic plasticity by regulating the cytosolic NADH/NAD + redox balance in postsynaptic neurons (Möller et al., 2021). Metabolic flux analysis further reveals that this lactate shuttle relies on AMP-activated protein kinase (AMPK)-mediated glycolytic activation in astrocytes and dynamic regulation of mitochondrial pyruvate carriers in neurons, providing both energy and signaling support for synaptic structural remodeling essential for memory formation (Yellen, 2018).

### 2.6 Therapeutic potential of targeting neuronal metabolism

Furthermore, the potential of metabolic interventions as therapeutic strategies is becoming a prominent research focus (Riera and Dillin, 2015). For instance, modulating glucose uptake and utilization in neurons via the AMPK signaling pathway, or enhancing mitochondrial metabolic flux through PGC-1α-dependent mitochondrial biogenesis, could offer neuroprotective effects under metabolic stress or neurodegenerative conditions (Lee and Silva, 2009). This approach may foster the creation of innovative therapies that specifically target neuronal insulin resistance (e.g., GLUT4 dysfunction) and enhance MCT2-mediated ketone uptake, thereby addressing the metabolic vulnerabilities of neurons and offering a promising pathway for intervening in neurodegenerative diseases (Antal et al., 2025).

### 2.7 Cutting-edge directions and future prospects of neuronal metabolism research

Looking ahead, integrating multi-omics approaches such as 4-dimensional fast data-independent acquisition (4D-FastDIA) quantitative proteomics and targeted metabolomics [e.g., dissecting how Danggui-Shaoyao-San regulates the glycogen synthase kinase 3 beta (GSK3β)/PGC1α signaling pathway to improve brain glucose uptake and mitochondrial complex function in AD models (Wu et al., 2024)] with computational modeling is unraveling systematic regulatory networks in neuronal energy metabolism, pinpointing critical nodes like pyruvate dehydrogenase complex phosphorylation and dynamic MCT2 transporter expression (Brown and Ransom, 2007). As the field advances toward personalized medicine, single-cell transcriptomics has revealed interindividual variability in neuronal glycolysis-oxidative phosphorylation balance (e.g., differential GLUT1/4 expression affecting substrate selection in APP/PS1 mice), offering new strategies for targeting AMPK-mediated astrocyte glycogenolysis to optimize neuronal energy supply (Wu et al., 2024).

In conclusion, while the fundamental aspects of neuronal energy metabolism are well-established, ongoing exploration of its complexities continues to reveal new layers of regulation and interaction. Future research will likely focus on the specific regulatory mechanisms of these metabolic processes in different neuronal populations, such as the lactate shuttle pathway and monocarboxylate transporter (MCT)-mediated interactions, how they are involved in the pathological processes of neurodegenerative diseases through key signaling pathways, and how to target specific metabolic enzymes and transporters for therapeutic intervention (Bélanger et al., 2011). This forward-looking perspective underscores that energy metabolism functions not merely as a support system for neuronal activity but as a pivotal regulator in brain health and disease through mechanisms such as mitochondrial ATP synthesis, calcium buffering, and oxidative stress response (Nicholls and Budd, 2000). Specifically, dysregulated neuronal energy metabolism can trigger apoptotic cascades via mitochondrial membrane potential collapse or abnormal pyruvate dehydrogenase complex phosphorylation, while targeting monocarboxylate transporter MCT2-mediated lactate shuttling or mitochondrial calcium uniporter (MCU) activity has emerged as a promising therapeutic strategy for neurodegenerative disorders.

## 3 Energy metabolism of astrocytes

### 3.1 Core characteristics and functions of astrocytic energy metabolism

Astrocytes, while requiring less energy than neurons, account for 5–15% of the brain’s total energy consumption (Attwell and Laughlin, 2001). In contrast to neurons with higher energy demands, astrocytes exhibit a greater capacity for glucose uptake, which exceeds their immediate energy requirements, as their glycolytic rate is several-fold higher than that of neurons (Barros et al., 2009). This excess glucose is utilized to synthesize glycogen, making.

astrocytes the brain’s sole glycogen reservoirs. Glycogen plays a crucial role during brain development, as neurons rely on astrocytes for energy during this critical stage. Astrocytes support neuronal energy metabolism through three primary pathways:

1. Glycogen Provision to Neurons: Astrocytes supply neurons with glycogen, which can be converted into glucose and used as an energy source.
2. Regulation of Glutamate Metabolism: Astrocytes modulate the levels of K^+^, Na^+^, and Ca2^+^ ions to regulate glutamate metabolism in neurons. This regulation is essential for maintaining neurotransmitter balance and ensuring proper neuronal function.
3. Lactate Delivery: Astrocytes convert glucose into lactate, which is then taken up by neurons and used as a fuel source.

These pathways highlight the critical role of astrocytes in supporting neuronal energy metabolism and overall brain function.

### 3.2 Regulatory mechanisms of glycolysis and oxidative phosphorylation in astrocytes

Astrocytes exhibit a greater glycolytic capacity relative to oxidative phosphorylation, supported by elevated Pfkfb3-driven fructose-2,6-bisphosphate production that accelerates glycolysis, while reduced pyruvate dehydrogenase expression restricts oxidative phosphorylation (Köhler et al., 2020). The elevated expression of Pfkfb3 in astrocytes facilitates the continuous production of fructose-2,6-bisphosphate, an allosteric activator of phosphofructokinase (PFK), which accelerates glycolysis (Bélanger et al., 2011). In contrast, astrocytes exhibit lower expression of pyruvate dehydrogenase (Laughton et al., 2007), which limits oxidative phosphorylation (Itoh et al., 2003) and reduces pyruvate utilization efficiency in the TCA cycle (Rahman et al., 2020). The concurrent production of NADH during pyruvate’s conversion to lactate also fosters a potent antioxidant environment, enabling astrocytes to sustain high glycolytic rates (Bélanger et al., 2011).

In addition to glycolysis, astrocytes store energy by synthesizing glycogen via Glc-6-P. Glycogen is indeed present in astrocytes within the brain (Briski et al., 2021), and under conditions of glucose deficiency or intense neural activity, astrocytic glycogen can be rapidly broken down via glycolysis to produce lactate (Brown and Ransom, 2007). This process, akin to glycolysis, is less prominent in neurons (Vilchez et al., 2007).

### 3.3 Metabolic crosstalk and adaptive regulation between astrocytes and neurons

As research into astrocytic energy metabolism progresses, several emerging trends are reshaping our understanding of the crucial roles astrocytes play in brain function. One significant area of interest is the interaction between astrocytes and neurons, particularly how astrocytes respond to neuronal signals to regulate their metabolic output. Recent studies suggest that astrocytes can dynamically adjust their metabolic pathways according to the demands of surrounding neurons. In response to neuronal activity, they modulate glycogen breakdown, glucose uptake, and lactate production through calcium-dependent signaling pathways, such as the release of glutamate which is triggered by elevated intracellular calcium levels (Chen et al., 2023). This crosstalk underscores the adaptability of astrocytes, positioning them as active participants in brain energy metabolism rather than merely passive supporters (Verkhratsky and Nedergaard, 2018).

### 3.4 Astrocytic metabolic reprogramming and neurodegenerative diseases

Another trend is the exploration of astrocytic metabolism in neurodegenerative diseases (Bélanger et al., 2011). Increasing evidence suggests that astrocytes may undergo metabolic reprogramming in conditions such as Alzheimer’s and Parkinson’s diseases. In these contexts, astrocytes may shift from a neuroprotective role to one that exacerbates neuronal dysfunction, potentially due to downregulated expression of lactate dehydrogenase A (LDHA), a key glycolytic enzyme, which impairs lactate shuttling by inhibiting large conductance Ca^2+^ activated potassium channel (BK channel)-mediated neuronal hyperpolarization (Yao et al., 2023). Understanding these molecular changes could reveal novel therapeutic targets aimed at restoring normal astrocytic function and protecting neurons from degeneration (Lalo et al., 2021).

### 3.5 Technological advances in astrocytic metabolism research

Furthermore, advances in imaging and metabolic tracing techniques enable researchers to observe real-time astrocyte-neuron metabolic interactions, such as astrocytes taking up glutamate via transporters and converting it to glutamine for neurons, providing new insights into how these cells coordinate to maintain brain homeostasis (Schousboe et al., 1997). These techniques are expected to clarify the specific mechanisms by which astrocytes sense and meet neuronal energy demands (such as through the monocarboxylate transporter-mediated astrocyte-neuron lactate shuttle), potentially revealing new therapeutic targets in brain metabolism (Yang et al., 2024).

### 3.6 Therapeutic potential of targeting astrocytic metabolism

Looking ahead, the potential of targeting astrocytic metabolism as a therapeutic strategy is garnering increased attention (Sun et al., 2022). By modulating key metabolic pathways in astrocytes, it may be possible to enhance their neuroprotective functions or prevent their transition to a harmful state in neurodegenerative diseases (Bak et al., 2018). For instance, enhancing astrocytic glycogen storage and promoting lactate production through the activation of glycogen phosphorylase and monocarboxylate transporters (MCTs) could ensure a stable energy supply for neurons during high-frequency firing or ischemic stress (Simard and Nedergaard, 2004).

### 3.7 Cutting-edge directions and future prospects of astrocytic metabolism research

In conclusion, while astrocytes have long been recognized for their supportive roles in the brain, current research is increasingly highlighting their active participation in brain energy metabolism and their potential involvement in neurological disorders. As we continue to unravel the complexities of astrocytic metabolism, these cells may emerge as crucial targets for interventions aimed at maintaining brain health and combating neurodegenerative diseases. This forward-looking perspective underscores the importance of continued research into the dynamic and multifaceted roles of astrocytes in the brain.

## 4 Relationship between astrocytes and neuronal energy metabolism

### 4.1 General metabolic synergy between astrocytes and neurons

Astrocytes not only sustain their own physiological activities but also provide crucial energy support to neurons, regulating synapse formation and information transmission. The energy metabolism of astrocytes is closely intertwined with that of neurons, facilitating a functional synergy between the two cell types. For example, neuronal activation is consistently associated with a 3–5 fold increase in astrocytic glucose uptake, as demonstrated by [^3^H]2DG autoradiography (Pellerin and Magistretti, 1994). During synaptic activity, astrocytes rapidly clear synaptic glutamate via Na^+^-dependent transporters (GLT-1/GLAST), activating the Na^+^/K^+^-ATPase which establishes a Na^+^ gradient driving GLUT1/3-mediated glucose uptake. This process culminates in lactate production via LDHB (EC50 = 80 μM), as confirmed by pharmacological inhibition of glutamate transporters (THA/L-CCG III) and Na^+^ substitution experiments.

### 4.2 Astrocyte–neuron lactate shuttle: mechanisms, signaling functions and controversies

Beyond binding to postsynaptic membrane glutamate receptors to generate postsynaptic currents or initiate cell signaling pathways, glutamate released into the synaptic space by the neuronal presynaptic membrane can also be taken up by astrocytes through glutamate transporters. This glutamate uptake in astrocytes requires the energy generated by the entry of Na^+^ ions into the astrocytes through the cis-electrochemical gradient. These Na^+^ ions are then actively transported out of the cell via the Na^+^/K^+^-ATPase enzyme. This increased ATP consumption in astrocytes leads to greater glucose uptake, mediated by an increase in glucose transporter production, which in turn boosts glycolysis and lactate production via LDHA.

**Figure 1:**
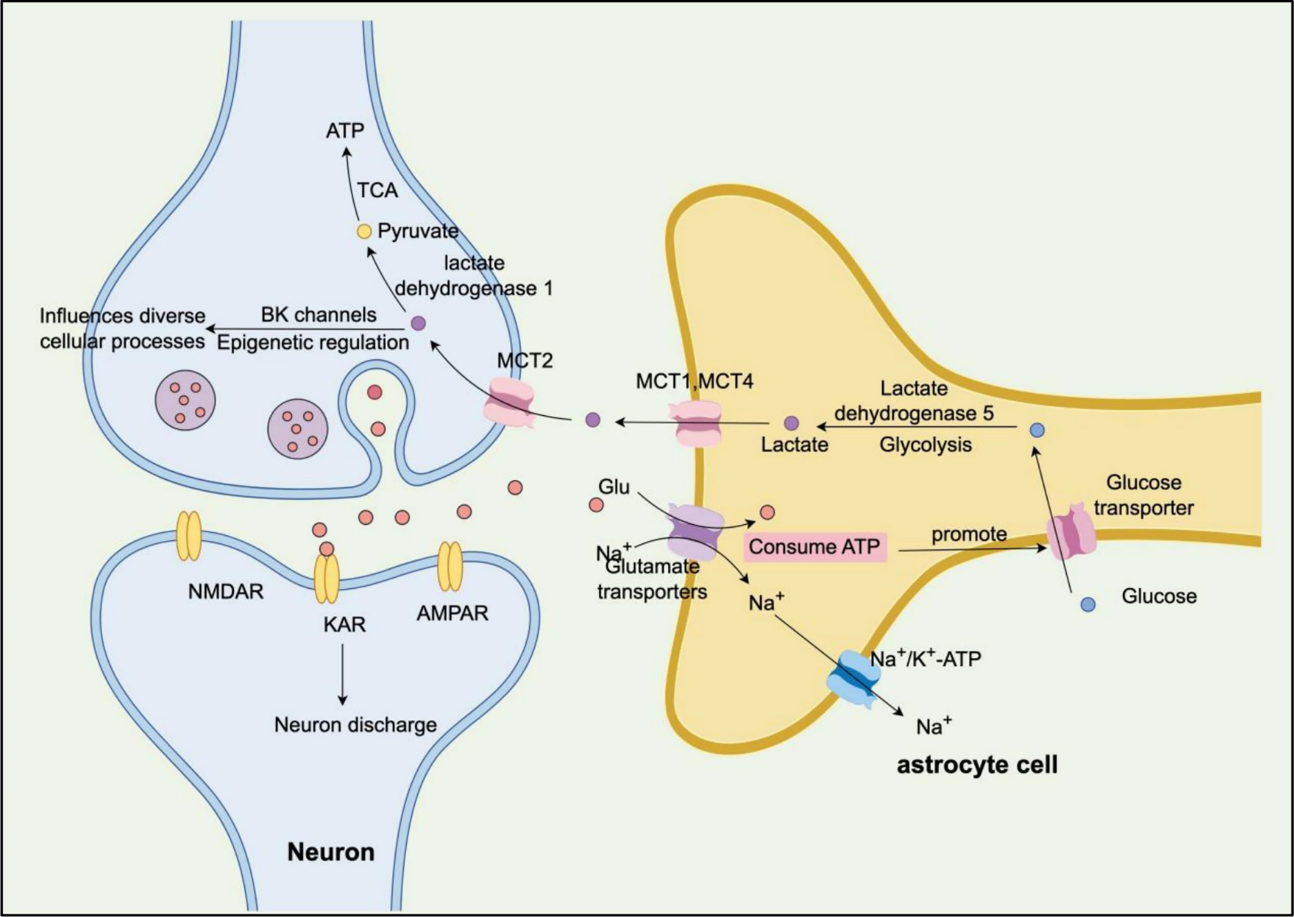
Schematic illustration of the astrocyte–neuron lactate shuttle (Brooks, 2018). This model depicts how glutamate uptake by astrocytes stimulates glycolysis and lactate production, which is then transported to neurons via monocarboxylate transporters (MCTs) to serve as an oxidative energy substrate (by Figdraw).

The lactate produced by glycolysis is then transported out of astrocytes through monocarboxylate transporters (MCT1 and MCT4) and taken up by neurons via MCT2 (Figure 1). In neurons, lactate is converted into pyruvate by lactate dehydrogenase 1, which then enters the TCA cycle and undergoes oxidative phosphorylation, resulting in the production of significant amounts of ATP to meet the energy demands of normal physiological activities.

Lactate also plays a role in enhancing neuronal excitability in the prefrontal cortex by modulating BK channels, specifically reducing the level of fast afterhyperpolarization (fAHP) during action potentials. The lactate signaling pathway primarily targets ATP-sensitive potassium (K-ATP) channels to regulate neuronal firing, with these channels being a key target for lactate signaling (Karagiannis et al., 2021). However, the precise molecular mechanisms remain unclear, with emerging evidence suggesting lactate may indirectly modulate BK channel function via metabolic-epigenetic crosstalk, such as activating Sirtuin 1 (SIRT1) deacetylase through NAD+/NADH redox modulation or altering channel phosphorylation via AMPK signaling, thereby influencing potassium current properties (Shipston and Tian, 2016; Yu et al., 2018; Li B. et al., 2020). Indeed, lactate acts as an epigenetic regulator by affecting histones, proteins that are critical in the packaging and organization of DNA, thereby influencing gene transcription and expression.

While lactate’s BK channel regulation remains unclear, its epigenetic role is revealed: lactate modifies histone lysine with 28 lactylation sites (e.g., H3K18la) in human/mouse cells. In M1 macrophages, elevated lactate induces Arg1 via lactylation, differing from acetylation dynamics. This offers a new path for neuro-glial metabolic crosstalk; future studies could focus on lactylation-mediated microglial cytokine regulation (Zhang et al., 2019).

It is crucial to acknowledge that the ANLS hypothesis, while supported by a substantial body of evidence as detailed above, remains a subject of active debate within the field. Critics point to methodological challenges and alternative interpretations of *in vivo* data. A prominent alternative viewpoint argues that the increased glucose uptake observed during neural activation is primarily metabolized via “aerobic glycolysis” (non-oxidative glucose consumption) (Fox et al., 1988) within the activated tissue itself, producing lactate that is largely released rather than shuttled to neurons. This perspective is often supported by neuroimaging studies (e.g., BOLD-fMRI) that report a mismatch between increases in glucose utilization (CMRglc) and oxygen consumption (CMRO₂) during brain activation. Proponents of this view suggest that neuronal energy demands during activation are met directly by glucose or other mechanisms, minimizing the quantitative importance of an intercellular lactate shuttle (Dienel, 2012, 2017).

A critical point of contention lies in the interpretation of the CMRO₂ measurements underlying this debate. The “non-oxidative” conclusion heavily depends on methods like BOLD-fMRI, which infer oxygen consumption from changes in vascular deoxyhemoglobin. A growing critique posits that such methods may not fully capture instantaneous oxygen consumption from pre-existing stores within the brain tissue itself (the parenchyma) (Schurr, 2025). This perspective is strengthened by recent findings demonstrating that lipid-rich structures, most notably the myelin sheaths that envelop axons, can act as significant oxygen reservoirs. Computational and biophysical models suggest myelin has a high oxygen capacitance and can temporarily supply oxygen to adjacent axons during increased metabolic demand (Morelli and Scholkmann, 2023; Vervust et al., 2025). Therefore, the observed hemodynamic uncoupling may reflect, in part, the initial utilization of these local oxygen pools rather than a true lack of oxidative metabolism (Schurr, 2025). The resolution of this debate likely hinges on technical advancements that allow direct, compartment-specific measurement of metabolic fluxes and oxygen dynamics in the tissue parenchyma.

Advances in astrocyte-neuron metabolic interaction research are uncovering mechanisms by which these cells maintain brain homeostasis. A pivotal approach employs cutting-edge imaging techniques like two-photon microscopy and fluorescence lifetime imaging, enabling real-time visualization of metabolic exchanges—such as lactate shuttle dynamics during synaptic activity. These methods reveal that astrocyte Ca^2+^ elevations, triggered by mechanical or electrical stimuli, drive glutamate release to modulate neuronal activity via ionotropic (iGluR) or metabotropic (mGluR) receptors, as seen in hippocampal slices where astrocyte Ca^2+^ waves inhibit or enhance presynaptic neurotransmission, highlighting context-dependent metabolic coupling in physiological and stress conditions (Chen et al., 2023). These technologies reveal the dynamic astrocyte-neuron lactate shuttle: during learning/memory, astrocytic glycogen-derived lactate transported via MCTs promotes Arc/cAMP response element-binding protein (CREB)-dependent synaptic plasticity; under stress, lactate supports neuronal KATP channels via mitochondrial oxidation, highlighting adaptive metabolic coupling (Bélanger et al., 2011).

Another area of increasing interest is the signaling role of astrocytic lactate beyond its role as an energy substrate: emerging evidence demonstrates that lactate induces histone lysine lactylation (e.g., H3K18la), activating transcription factors like Klf4 to regulate genes associated with synaptic plasticity (e.g., Arc, CREB), thereby contributing to epigenetic remodeling during long-term memory formation—a mechanism that underscores metabolic signaling as a target for cognitive enhancement (Yao et al., 2023). This concept opens up exciting possibilities for new therapeutic strategies targeting metabolic pathways to enhance cognitive functions, especially in aging and neurodegenerative diseases (Lalo et al., 2021).

Furthermore, the link between astrocytic metabolism and neurodegeneration is gaining traction: during brain aging or degenerative processes, the efficiency of astrocyte-neuron metabolic coupling—critical for glutamate homeostasis via transporters like GLT-1 and GLAST—declines, leading to reduced glutamate clearance, extracellular accumulation, and NMDA receptor-mediated excitotoxicity, which drives neuronal dysfunction. In Alzheimer’s disease, for example, downregulation of GLT-1 correlates with synaptic damage and exacerbates disease progression (Schousboe et al., 1997). In Alzheimer’s disease, astrocytic dysfunction impairs glutamate clearance via downregulated GLT-1/GLAST and reduces lactate production through decreased lactate dehydrogenase A (LDHA) activity, leading to neuronal energy deficits and exacerbated NMDA receptor excitotoxicity. Targeting astrocyte-mediated glutamate transport (e.g., enhancing GLT-1 function) or lactate shuttling (e.g., activating MCT1/2 transporters) represents a promising therapeutic strategy to preserve neuronal bioenergetics and slow disease progression (Yang et al., 2024).

Looking ahead, research is also beginning to explore how systemic metabolic states, such as those influenced by diet and exercise, affect astrocyte-neuron interactions (Sun et al., 2022). There is growing interest in how metabolic interventions, like ketogenic diets or intermittent fasting, might enhance brain energy metabolism by modulating astrocytic functions (Bak et al., 2018). These approaches could offer new ways to support cognitive health and combat neurodegenerative disorders (Simard and Nedergaard, 2004).

In conclusion, while significant progress has been made in understanding the metabolic interplay between astrocytes and neurons, many questions remain unanswered. Future research will likely focus on elucidating the molecular mechanisms underlying astrocyte-mediated modulation of neuronal activity, the role of lactate as a signaling molecule, and how these processes are altered in neurodegenerative diseases. The continued integration of advanced imaging, molecular biology, and metabolic studies will be crucial in uncovering the full extent of astrocyte-neuron metabolic cooperation and its implications for brain health and disease (Verkhratsky and Nedergaard, 2018). This forward-looking perspective highlights the potential of targeting astrocytic metabolism as a novel therapeutic strategy in the fight against neurodegenerative diseases and cognitive decline (Simard and Nedergaard, 2004).

### 4.3 Astrocyte– neuronal glutathione (GSH) shuttle: antioxidant defense and neurodegenerative diseases

**Figure 2:**
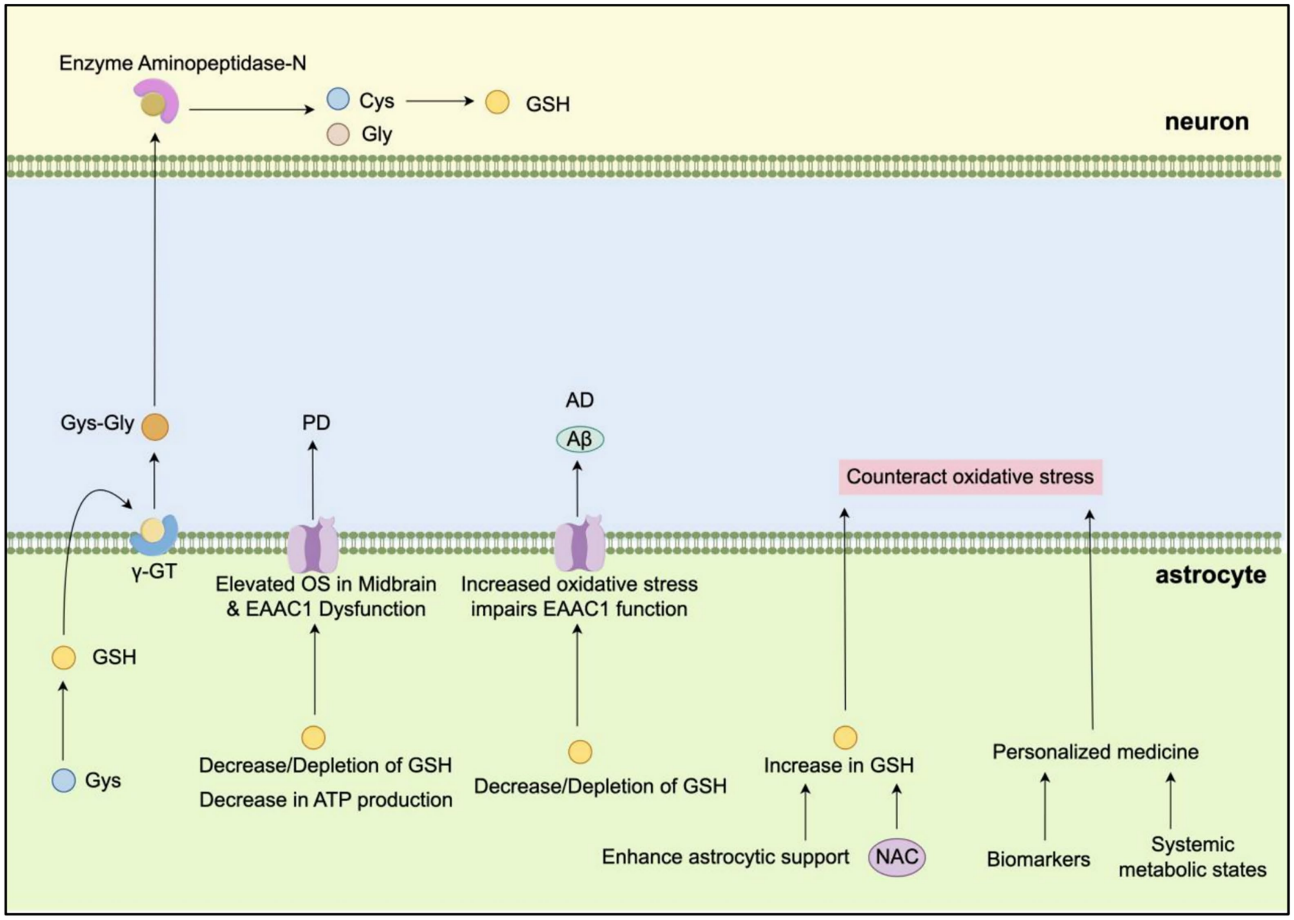
Schematic illustration of the astrocyte–neuron glutathione (GSH) shuttle (Dringen, 2000). This model highlights the synthesis and release of GSH by astrocytes, its extracellular processing, and subsequent uptake and utilization by neurons for antioxidant defense (by Figdraw).

Glutathione (GSH) is a critical antioxidant in the brain, playing a vital role in protecting neurons from oxidative damage by scavenging reactive oxygen species (ROS). Astrocytes efficiently synthesize GSH using cysteine (Cys) and release it extracellularly (Figure 2). Once outside the cell, GSH is cleaved into Cys-Gly by the enzyme *γ*-glutamyl transpeptidase (γ-GT), which is located on the astrocytic cell membrane. Neurons then take up Cys-Gly and convert it into Cys and Gly via the enzyme aminopeptidase-N. This process enables neurons to use Cys for synthesizing their own GSH, which is essential for defending against oxidative stress.

Numerous studies have highlighted the significance of oxidative stress and reduced GSH levels as major risk factors for neurodegenerative lesions in the central nervous system (CNS) across various neurological diseases (Dringen, 2000). GSH levels in the brain tend to decline in age-related neurodegenerative diseases, such as amyotrophic lateral sclerosis (ALS), Parkinson’s disease (PD), and Alzheimer’s disease (AD) (Aoyama and Nakaki, 2013). Postmortem analyses of brains from PD patients have shown a marked decrease in GSH levels, particularly in the substantia nigra of the midbrain (Rae and Williams, 2017). This reduction in intracellular GSH concentrations, combined with diminished ATP production, has been linked to increased vulnerability of dopaminergic neurons in PD, as observed in both *in vitro* and *in vivo* studies (Sian et al., 1994). In the MPTP mouse model of PD, GSH depletion resulted in elevated oxidative stress in the midbrain and dysfunction of the Excitatory amino acid carrier 1(EAAC1) transporter (Zeevalk et al., 1998). These findings suggest that a decline in neuronal GSH levels is a critical event preceding the onset of PD.

In AD, the abnormal aggregation of amyloid-beta (Aβ) protein contributes to increased oxidative stress, which is believed to play a central role in disease onset. Aβ has been shown to impair EAAC1 transporter function, thereby inhibiting cysteine uptake and further exacerbating oxidative stress (Aoyama et al., 2008). These observations emphasize the importance of maintaining optimal GSH levels and minimizing oxidative stress to preserve neuronal health and prevent the progression of neurodegenerative diseases. However, further research is needed to fully elucidate the underlying mechanisms and to develop targeted therapeutic interventions.

In patients with AD or mild cognitive impairment, proton magnetic resonance spectroscopy (1H-MRS) has revealed significantly lower hippocampal GSH levels compared to healthy elderly controls (Hodgson et al., 2013), suggesting that GSH depletion may play a role in AD pathogenesis. Similarly, oxidative stress is a hallmark of ALS (Mandal et al., 2015). In clinical studies where brain glutathione (GSH) levels were quantified using standard J-edited spin-echo differential magnetic resonance spectroscopy (MRS), results indicate that demonstrating decreased GSH levels in the brains of ALS patients compared to age-matched healthy volunteers, particularly in the motor cortex (Aoyama et al., 2000; Weiduschat et al., 2014). These findings further underscore the involvement of GSH in the development and progression of ALS.

Table 2 summarizes the key metabolic alterations observed in neurons, astrocytes, and microglia across major neurodegenerative diseases.

**Table 2:** Metabolic alterations in neurodegenerative diseases.

Disease	Abnormal metabolism of neurons	Metabolic abnormalities of astrocytes	Metabolic abnormalities of microglia
AD	Impaired EAAC1 function Decreased GSH Energy deficiency and oxidative stress PDK4 inhibiting PDH PKM2/PKM1 switch	LDHA downregulation Reduced lactate production GLT-1/GLAST downregulation Glutamate clearance impairment Abnormal glucose metabolism Glycolytic enzyme metabolism	Promote neuroinflammation Affect synaptic plasticity
PD	Decreased GSH level Impaired EAAC1 function Energy vulnerability of dopaminergic neurons Increased oxidative stress	Mitochondrial dysfunction α-synuclein clearance impairment	Participate in inflammation and neuronal damage
ALS	Oxidative stress GSH decline	Abnormal glutamate metabolism Decreased expression of EAAT2 leading to glutamate excitotoxicity	Activation promotes inflammation

GSH’s pivotal role in protecting neurons from oxidative damage has sparked significant interest in its potential as a therapeutic target for neurodegenerative diseases. As understanding of oxidative stress in neurodegeneration deepens, current research is increasingly focusing on the mechanisms that drive GSH depletion and the broader implications of oxidative imbalance in the brain.

A key emerging trend is the exploration of GSH-boosting strategies as potential therapeutic interventions. For example, research is investigating the use of GSH precursors, such as N-acetylcysteine (NAC), to enhance intracellular GSH levels and counteract oxidative stress in neurodegenerative conditions (Arakawa and Ito, 2007). Additionally, novel blood–brain barrier-penetrant compounds (e.g., lipoic acid derivatives, GSH precursor transporter agonists) are being developed to boost CNS GSH levels by activating glutamate cysteine ligase in neurons or enhancing precursor uptake via OATP at the blood–brain barrier, thereby restoring neuronal antioxidant capacity and protecting against tau oxidation in AD and dopaminergic neuron loss in PD (Sun and Chen, 1998).

In addition to the GSH system, the brain employs other endogenous antioxidant mechanisms. For instance, lactate plays a dual role in aerobic metabolism: it serves not only as an important energy substrate but also contributes to antioxidant defense. The concomitant production of reduced NADH during the mitochondrial conversion of lactate to pyruvate can directly scavenge ROS. Schurr and Gozal demonstrated that during neuronal activation, NADH—produced via mLDH—acts as an endogenous ROS scavenger, neutralizing oxidative stress induced by glutamate excitotoxicity and thereby conferring neuroprotection (Schurr and Gozal, 2011). This lactate-NADH antioxidant mechanism operates in concert with the GSH system, collectively maintaining neuronal redox balance under conditions of activation or metabolic stress. Its role may be particularly significant during early metabolic disturbances in neurodegenerative diseases.

Another area of growing interest is the role of astrocyte-neuron interactions in maintaining GSH homeostasis: astrocytes synthesize GSH by importing cystine via the cystine/glutamate antiporter (Sxc^−^) and glutamine synthetase, releasing GSH directly through pannexin-1 channels or gap junctions (Cx43), while regulating extracellular glutamate levels via excitatory amino acid transporter 1/2(EAAT1/2) transporters to sustain neuronal GSH precursor supply, thereby mediating neuroprotection under oxidative stress (Verkhratsky and Nedergaard, 2018). Future research is likely to focus on enhancing astrocytic support for neuronal GSH levels, particularly in aging and neurodegeneration: strategies may include activating Nrf2-mediated antioxidant responses to upregulate cystine/glutamate antiporter (Sxc^−^) and glutamine synthetase in astrocytes, or targeting AMPK/SIRT1 pathways to facilitate GSH precursor transfer to neurons. Elucidating such signaling mechanisms could uncover novel therapeutic targets by leveraging astrocyte-neuron metabolic crosstalk (Dringen, 2000).

Moreover, the potential of personalized medicine in managing oxidative stress-related neurodegeneration is gaining momentum: genetic variants in the Nrf2 signaling pathway or SIRT1-mediated deacetylation affecting astrocytic GSH synthesis (e.g., regulating glutamate cysteine ligase catalytic subunit expression), combined with biomarkers like individual GSH levels and Nrf2 activity, could enable tailored interventions. For instance, activating AMPK/SIRT1 pathways to enhance neuronal GSH precursor supply or targeting Nrf2-dependent antioxidant gene expression, particularly addressing SIRT1 hypofunction in Alzheimer’s disease, may optimize neuroprotective outcomes by addressing patient-specific metabolic vulnerabilities (Simon et al., 2020). The field is also exploring the link between metabolic health and GSH levels: emerging evidence indicates that systemic metabolic states, including dietary patterns (e.g., fiber-rich diets enhancing short-chain fatty acids) and gut microbiota, modulate brain GSH levels via the gut-brain axis. Mechanisms involve Nrf2-dependent upregulation of glutamate cysteine ligase in astrocytes, thereby balancing redox status and oxidative stress (Sharon et al., 2016). This opens up possibilities for lifestyle interventions to modulate GSH levels and support brain health: ketogenic diets, by generating ketone bodies like β-hydroxybutyrate, activate astrocytic Nrf2 signaling to upregulate glutamate cysteine ligase and enhance GSH synthesis; intermittent fasting may improve neuronal GSH precursor transport via AMPK/SIRT1 pathways. Investigating how such interventions affect brain GSH metabolism could reveal new strategies for neuroprotection (McDougall and Stewart, 2005). The neuroprotective potential of the ketogenic diet is closely linked to its induced metabolic remodeling: by generating ketone bodies like β-hydroxybutyrate, it activates astrocytic Nrf2 signaling to upregulate glutamate cysteine ligase, directly enhancing glutathione (GSH) synthesis and brain antioxidant capacity. Additionally, the diet modulates gut microbiota (e.g., enriching *Akkermansia muciniphila*), indirectly improving neuroinflammatory microenvironments via the gut-brain axis, offering new targets for interventions in neurodegenerative diseases through metabolic-microbial crosstalk (Dilmore et al., 2023). The neuroprotective effects of the ketogenic diet are closely linked to its induction of metabolic remodeling and protein modification: by increasing β-hydroxybutyrate levels, it specifically promotes β-hydroxybutyrylation of key tricarboxylic acid (TCA) cycle enzymes (such as citrate synthase [CS] and succinate-CoA ligase subunit alpha [SUCLG1]), enhancing enzymatic activity and ATP production to improve neuronal energy metabolism. Additionally, the diet reduces amyloid- β plaque deposition and microglial overactivation in Alzheimer’ s model mice, providing a novel mechanism for intervening in neurodegenerative diseases through regulation of metabolic enzyme modifications (Han et al., 2025). The neuroprotective effects of the ketogenic diet further extend to synaptic plasticity regulation: by increasing β-hydroxybutyrate (BHB) levels, it directly activates the ERK/CREB signaling pathway in the hippocampus, promoting brain-derived neurotrophic factor (BDNF) synthesis (especially in females) to restore impaired long-term potentiation (LTP) in Alzheimer’s model mice. Additionally, the diet reduces the expression of microglial activation markers (e.g., Iba1, CD11b), mitigating neuroinflammation, with its protective effects independent of amyloid- β plaque reduction, thus offering new targets for intervening in early cognitive dysfunction (Di Lucente et al., 2024).

While GSH has long been recognized as a key brain antioxidant, its role in neurodegenerative diseases is now being unraveled with mechanistic clarity: future research will likely focus on activating astrocytic Nrf2 signaling to enhance glutamate cysteine ligase (GCL)-mediated GSH synthesis, exploring the role of MCT1/2 transporters in neuronal GSH precursor supply, and dissecting how oxidative stress modulates microglial activation via pathways like NF-κB-dependent proinflammatory cytokine secretion (Sun et al., 2022). The continued integration of molecular biology, genetics, and systems biology approaches is pivotal to unraveling the full therapeutic potential of glutathione (GSH). This forward-looking perspective highlights that maintaining brain redox balance—by targeting astrocyte-neuron metabolic coupling and microglial oxidative stress pathways—represents a central strategy in combating neurodegenerative diseases (Aoyama, 2021).

## 5 Role of astrocytes in neuronal energy metabolism

Astrocytes are crucial for neuronal synapses, regulating their formation, function, and activity, and one way is by releasing gliotransmitters like ATP, as shown by GRABATP1.0 - based research revealing spontaneous ATP release that can modulate synaptic activity (Hatashita et al., 2023). For instance, astrocytes can significantly inhibit glutamate release by interacting with A1 receptors (A1R) and facilitating the breakdown of ATP into adenosine in the synaptic space. In experiments, the activation of astrocytes expressing ChR2 leads to a significant increase in extracellular ATP and adenosine concentrations. Adenosine binds to A1R, inhibiting the excitability of neurons and reducing glutamate release. This process involves the activation of receptors such as P2Y1, affecting the intracellular signaling pathways and ultimately achieving the inhibition of glutamate release (Li Y. et al., 2020). Beyond their influence on synaptic activity, astrocytes play a crucial role in regulating neuronal excitability by modulating energy metabolism.

By measuring insulin-induced Akt phosphorylation (pAkt) within a specific hypothalamic nucleus, an animal study revealed that specific hypothalamic nuclei are responsible for regulating satiety and feeding behavior. These nuclei can increase the glucose levels in the central nervous system through specific mechanisms. For example, they can promote the translocation of glucose transporters to the cell membrane, allowing more glucose to enter the extracellular environment. Once the glucose concentration rises, it binds to glucose—sensitive receptors on the neurons of the hypothalamic nuclei, triggering a series of intracellular signal transduction processes, thus enhancing the discharge frequency of these nuclei. This change affects insulin secretion from the pancreas and the uptake and metabolism of glucose in organs such as the liver and muscles through the neuroendocrine pathway, ultimately influencing blood glucose and insulin levels (Ladyman and Grattan, 2017). Astrocytes are central to this process: lactate produced by astrocytes is transported into neurons via monocarboxylate transporter MCT2, where lactate dehydrogenase B (LDHB) converts it to pyruvate. This conversion reduces the intracellular ATP/ADP ratio, inhibiting the opening of Kir6.1/SUR1-type K+-ATP channels in hypothalamic neurons, thereby increasing their firing frequency (Shi et al., 2024). This metabolic-electrophysiological coupling precisely modulates energy homeostasis and glucose metabolism by regulating the activity of hypothalamic satiety centers. Additionally, lactate from astrocytes can regulate the electrophysiological activity of orexin neurons. It acts through monocarboxylate transporters on orexin neurons, and this effect is mediated by ATP—sensitive potassium channels composed of Kir6.1 and SUR1 subunits, thus influencing wakefulness and energy homeostasis (Parsons and Hirasawa, 2010).

A patch—clamp study adopted a comprehensive strategy spanning molecular to physiological levels. It utilized inducible cell lines for initial validation, corroborated relevance in ex vivo and knockout models, and finally interrogated the underlying mechanisms using brain slice electrophysiology and pharmacology. And it showed that when both lactate and glucose are available, orexin neurons preferentially use lactate. They adjust their spontaneous firing frequency in response to lactate levels, and this process is mediated by ATP—sensitive potassium channels composed of Kir6.1 and SUR1 subunits. The spontaneous firing is significantly inhibited by monocarboxylate transporter (MCT) inhibitors. Pharmacological ablation of astrocytes disrupts the ability of orexin neurons to respond to glucose concentration changes, as orexin neurons rely on astrocyte - derived lactate. In the subfornical organ (SFO) that regulates salt uptake, the firing frequency of GABAergic neurons is affected by lactate from astrocytic glycolysis. In SFO astrocytes, the Nax channel interacts with the Na^+^/K^+^-ATPase. This interaction activates the Na^+^/K^+^-ATPase, which increases energy (ATP) consumption. To meet this heightened energy demand rapidly, astrocytes enhance glycolytic flux, leading to increased lactate production. The produced lactate then activates GABAergic neurons through monocarboxylate transporters, raising their firing rate (Shimizu et al., 2007).

In the rat hippocampus, the learning process significantly increases the levels of glycogen-derived lactate in astrocytes. This is because learning activates the glycogenolytic pathway in astrocytes, leading to more glycogen being converted into lactate. When the expression of MCT4 or MCT1 in astrocytes is disrupted, rats experience amnesia and impaired long - term potentiation (LTP). MCT4 and MCT1 are responsible for transporting lactate produced by astrocytes to the extracellular space. Disrupting their expression hinders the normal transport of lactate, affecting neuronal energy supply and signal transmission, thus disrupting LTP and memory formation. However, supplementing with L—lactate can restore these impaired functions because the supplemented lactate can directly provide energy for neurons, maintaining normal synaptic plasticity and memory—related physiological processes. Nevertheless, disrupting the expression of MCT2 also causes amnesia, but lactate or glucose supplementation fails to improve this situation. MCT2 is mainly responsible for transporting extracellular lactate into neurons. When its expression is disrupted, lactate cannot enter neurons normally, resulting in an insufficient neuronal energy supply. Even with the supplementation of lactate or glucose, the normal LTP and memory functions cannot be restored, which fully demonstrates that the transport of lactate to neurons is crucial for LTP and long - term memory formation (Matsui et al., 2017).

In the brain, the synthesis and breakdown of astrocyte glycogen are regulated by multiple factors. Norepinephrine binds to adrenergic receptors on astrocytes, affecting the activity of glycogen metabolism - related enzymes. Serotonin (5 - HT) binds to 5 - HT₂B receptors, increasing intracellular calcium levels via the Phospholipase C (PLC) pathway to stimulate glycogenolysis. Vasoactive intestinal peptides secreted by neurons elevate cAMP levels, activating PKA to regulate glycogen synthase and phosphorylase, thus promoting glycogen breakdown. Additionally, changes in local glucose concentration also impact glycogen synthesis and breakdown (Hertz et al., 2015; Briski et al., 2021). The glycogen synthesized by astrocytes plays a role in regulating the sleep cycle. During sleep, the brain’s energy demand changes. Astrocytic glycogen can be broken down into lactate, which is then shuttled to neurons. As shown in relevant research involving both adolescent and adult populations with type 1 diabetes, lactate can act as an energy substrate for neurons, maintaining their energy levels and influencing the sleep-wake cycle (Perfect, 2020; Ji et al., 2021). Furthermore, astrocytic glycogen metabolism is crucial for neural activity adjustments in response to blood loss or intense stimulation, especially in the white matter of the mouse brain (Brown et al., 2005). When blood loss or intense stimulation occurs, astrocytes break down glycogen into lactate. As demonstrated in relevant studies, lactate can be transported to neurons and serve as an energy substrate, maintaining the transmembrane ion gradients required for axons to conduct action potentials. This process helps to sustain neural activity. For example, in experiments on mouse optic nerves, when glycogen metabolism was inhibited, the compound action potential (CAP) failed more rapidly during high—intensity stimulation, indicating the importance of astrocytic glycogen metabolism in supporting neural function under such conditions. *In vitro* experiments show that astrocyte glycogen synthesis helps neurons synthesize and release glutamate neurotransmitters. Neurotransmitters like norepinephrine bind to astrocyte receptors, activating the cAMP pathway to promote glycogen synthesis. Glutamate is taken up by astrocytes for metabolism, providing energy for glycogen synthesis. The synthesized glycogen then supplies energy and reducing power for glutamate synthesis and release (Laureys et al., 2014; Beard et al., 2021).

As research on the role of astrocytes in neuronal energy metabolism continues to advance, several new trends are emerging in this field. One important trend is the focus on how astrocytic metabolism is dynamically regulated in response to neuronal activity. When neurons are active, they release neurotransmitters such as norepinephrine and vasoactive intestinal peptide. These neurotransmitters bind to corresponding receptors on astrocytes, activating signaling pathways like cAMP. For example, norepinephrine binds to β-adrenergic receptors, activating adenylate cyclase through Gs protein, which increases the intracellular cAMP concentration and then activates protein kinase A (PKA). PKA can phosphorylate glycogen phosphorylase, promoting glycogen breakdown and providing energy for neuronal activity. Researchers are increasingly using advanced imaging techniques such as two—photon microscopy to observe real - time changes in astrocyte metabolism during neural activity. This allows for a deeper understanding of the specific mechanisms by which astrocytes support synaptic function and maintain overall brain energy homeostasis (Chen et al., 2023).

Another emerging area of interest is the impact of systemic metabolic states on astrocyte - neuron interactions. Mounting evidence shows that metabolic conditions like obesity and diabetes, along with dietary interventions, can affect astrocytic metabolism through multiple mechanisms. For example, in obesity, increased leptin levels bind to leptin receptors on astrocytes, activating the JAK/STAT signaling pathway. This leads to changes in astrocytic glucose metabolism and glutamate transport, which in turn influence neuronal function by altering synaptic transmission and energy supply. Understanding these connections could pave the way for novel therapeutic strategies. By modulating astrocytic activity through targeted regulation of relevant signaling pathways, it may be possible to improve cognitive function and safeguard against neurodegenerative diseases (Verkhratsky and Nedergaard, 2018).

Moreover, the role of astrocytes in neurodegenerative diseases has emerged as a crucial research focus. In Alzheimer’s disease (AD), for example, astrocytes show abnormal glucose metabolism and reduced lactate production. This is related to the downregulation of glycolytic enzymes, which may lead to insufficient energy supply for neurons and abnormal accumulation of amyloid-β. In Parkinson’s disease (PD), astrocytic mitochondrial dysfunction disrupts the clearance of *α* -synuclein through the ubiquitin - proteasome system and autophagy pathways, promoting the formation of Lewy bodies. In amyotrophic lateral sclerosis (ALS), astrocytes exhibit abnormal glutamate metabolism, with reduced expression of glutamate transporters like EAAT2. This causes glutamate excitotoxicity, contributing to motor neuron degeneration. Future research is likely to focus on strategies to restore normal astrocytic metabolism, such as targeting specific metabolic enzymes or transporters, which may potentially slow or halt the progression of these diseases (Yao et al., 2023).

A particularly promising research direction is to explore metabolic interventions to enhance the supportive effect of astrocytes on neurons. Take the ketogenic diet and caloric restriction as examples. The ketogenic diet can increase the level of ketone bodies in the body, among which β-hydroxybutyrate (BHB) can act on astrocytes. In a mouse model of multiple sclerosis, BHB can activate the Notch signaling pathway, improve stem cell homeostasis, and promote the survival and regeneration of neurons. At the same time, BHB can also inhibit the inflammatory response, reduce the secretion of pro-inflammatory cytokines such as IL-1β, IL-6, and TNF-*α*, and decrease the activation levels of astrocytes and microglia, creating a more favorable survival environment for neurons. Caloric restriction may regulate the energy sensors in cells, such as the AMPK signaling pathway, affecting the metabolism of astrocytes and enabling them to produce more substances beneficial to neurons, such as neurotrophic factors, thereby protecting neurons from degeneration. These dietary strategies are expected to be further developed and become an important part of a comprehensive therapy for neurodegenerative diseases (Sun et al., 2022).

Recently, the role of astrocytes in sleep regulation has attracted increasing attention. Research has found that during sleep, astrocytic glycogen metabolism is crucial for maintaining brain energy levels. During wakefulness, especially sleep deprivation, brain glycogen levels decline, while they are replenished during sleep. This process involves the regulation of intracellular signaling pathways such as cAMP and Ca^2+^, which can activate glycogen phosphorylase and promote glycogen breakdown to provide energy for neurons. Sufficient brain energy supply is conducive to memory consolidation and the normal functioning of cognitive abilities, as memory consolidation requires energy to support signal transmission between neurons and the adjustment of synaptic plasticity. In - depth exploration of the mechanisms by which astrocytes participate in sleep - related processes may provide new methods for treating sleep disorders and improving cognitive health. For example, drugs that regulate astrocytic glycogen metabolism could be developed to correct the brain energy imbalance in patients with sleep disorders (Bak et al., 2018).

In conclusion, while much has been learned about the role of astrocytes in neuronal energy metabolism, ongoing research continues to reveal the complexity and importance of these cells in brain function. Future studies will likely focus on the molecular mechanisms underlying astrocyte-neuron metabolic interactions, the impact of systemic metabolic states on brain health, and the potential of targeting astrocytic metabolism in neurodegenerative disease therapies. This forward-looking perspective highlights the critical role of astrocytes in maintaining brain energy homeostasis and suggests new therapeutic avenues for promoting brain health and resilience.

## 6 Core characteristics, distribution, and phenotypic plasticity of microglia

### 6.1 Microglial heterogeneity, activation signaling, and biphasic regulation in neurodegeneration

**Figure 3:**
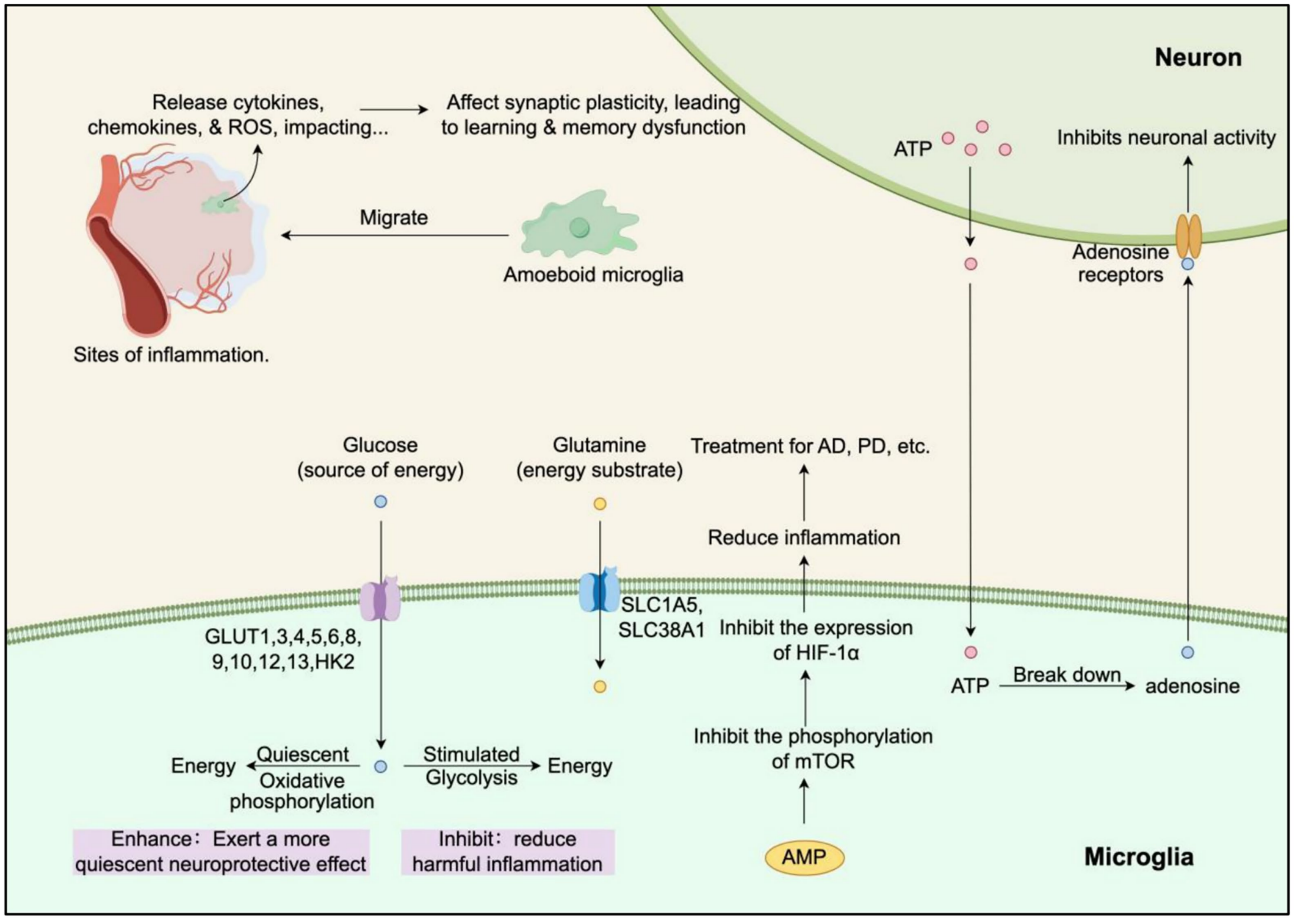
Effects of microglia metabolism on neurons and diseases (by Figdraw).

Microglia, which constitute 10–15% of brain cells, play a critical role in the innate immune response and neuroplasticity (Figure 3). The density of microglia varies across brain regions, with the highest densities found in the cortex, striatum, corpus callosum, hippocampus, subventricular zone, and rostral migratory stream. Intermediate densities are observed in the olfactory bulb, thalamus, hypothalamus, midbrain, and brainstem, while the cerebellum has the lowest microglial density (Campos-Torres et al., 2005; Stratoulias et al., 2019; Tan et al., 2020). Microglia exhibit distinct gene expression profiles and functional heterogeneity, maintaining a ramified morphology at rest via CSF1R signaling. Upon activation, CX3CR1-mediated chemotaxis induces a shift to an amoeboid shape, accompanied by upregulation of TREM2-dependent phagocytic receptors and P2X7R activation, triggering dynamic secretion of pro-inflammatory cytokines (e.g., IL-1β, TNF-α) or anti-inflammatory factors (e.g., IL-10, TGF-β). This phenotypic plasticity manifests as biphasic regulation in neurodegeneration: early protective phagocytosis of Aβ plaques versus late-stage pro-inflammatory exacerbation, modulated by microenvironmental signals like local ATP concentration and neuron-secreted IL-34 (Eyo et al., 2013; Colonna and Butovsky, 2017; Tan et al., 2020).

### 6.2 Energy substrates and metabolic characteristics of microglia

Glucose is the primary energy source for microglial survival and function, with hypoxic glucose deprivation leading to microglial cell death (Kacimi et al., 2011; Kalsbeek et al., 2016). Microglia express multiple glucose transporters, including GLUT1, 3, 4, 5, 6, 8, 9, 10, 12, and 13, as well as high levels of hexokinase 2 (HK2), which collectively ensure sufficient glucose influx for their metabolic needs (Baik et al., 2019; Wang et al., 2019). Among these, GLUT1 is the most highly expressed in microglia and plays a key role in glucose uptake, especially under inflammatory conditions (Baik et al., 2019). In addition to glucose, microglia utilize glutamine as an energy substrate, facilitated by the high expression of glutamine transporters SLC1A5 and SLC38A1 (Liu et al., 2018).

### 6.3 Metabolic regulation of microglial functional phenotypes

The energy metabolism of microglia varies depending on their activation state. Quiescent microglia rely on oxidative phosphorylation of glucose to meet their energy demands. However, when microglia are stimulated, they shift from oxidative phosphorylation to glycolysis (Cheng et al., 2014). Although glycolysis produces less ATP compared to mitochondrial respiration, it generates lactate more rapidly, which supports energy-intensive processes such as proliferation, migration, and secretion (Werneburg et al., 2017; Elmore et al., 2018). AMP-activated protein kinase (AMPK), a sensor for AMP and ADP, inhibits mechanistic target of rapamycin (mTOR) phosphorylation, thereby suppressing the expression of hypoxia-inducible factor 1-alpha (HIF-1α), the master transcriptional regulator of glycolysis (Peek et al., 2017). Inhibition of the mTOR pathway significantly reduces the inflammatory response in microglia, highlighting how cellular metabolic pathways, particularly the mTOR-HIF-1 pathway, can influence the functional phenotype of microglia (Cheng et al., 2014).

## 7 Impact of microglial metabolism on neurons

### 7.1 Physiological regulation of neurons by microglia

Microglia, often referred to as the brain’s macrophages, play a vital role in regulating brain function. They perform various essential tasks, including clearing dead neurons, eliminating non-functional synapses, and producing ligands that support neuronal survival, all of which contribute to the overall regulation of brain function (Saw et al., 2020). Under normal physiological conditions, motile microglia continuously monitor their microenvironment. Through interactions with neurons, microglia regulate crucial processes such as neuronal activity, synapse formation and survival, and the remodeling of presynaptic circuit (Badimon et al., 2020; Saw et al., 2020; Sun et al., 2020).

Neuronal activity plays an active role in modulating microglial function, particularly in synapse pruning, which ultimately influences synaptic plasticity (Kim et al., 2020). Synaptic plasticity is the central nervous system’s ability to modify synaptic and neural connections in response to synaptic activity, sensory input, and motor experiences. Microglia are key players in this regulation, affecting both long-term potentiation (LTP) and long-term depression (LTD), which are fundamental mechanisms underlying synaptic plasticity (Raghuraman et al., 2019; Kim et al., 2020).

Moreover, in the adult brain, microglia promote dendritic spine formation through the secretion of brain-derived neurotrophic factor (BDNF). Remarkably, studies have shown that microglial regeneration in humans is feasible. Replantation of microglia in the brains of individuals with Alzheimer’s disease (AD) has been found to restore synaptic density and neurosynaptic plasticity, thereby alleviating AD-related learning and memory impairments (Wang W. et al., 2023). These findings underscore the intricate relationship between microglia and neurons, highlighting the critical role of microglial metabolism in maintaining optimal brain function and offering promising therapeutic potential for neurodegenerative diseases like AD.

In addition to regulating neuronal activity, microglia are adept at sensing and responding to neural activation. Similar to inhibitory neurons, microglia can detect excessive neuronal activity and inhibit it, thereby protecting the brain from disease (Akiyoshi et al., 2018). When neurons are activated, they release ATP molecules that microglia can sense. Microglia then break down ATP into adenosine, which binds to adenosine receptors on the surface of active neurons. This binding effectively inhibits neuronal activity, preventing overactivation. This negative feedback mechanism is crucial for regulating neuronal activity, as excessive hyperactivity can lead to neuronal death, synaptic loss, and reduced plasticity (Badimon et al., 2020).

### 7.2 Pathological roles of aberrant microglial activation

Microglia also play a significant role in responding to brain injury, infection, or neuroinflammation. Upon activation, microglia undergo morphological changes, becoming amoeboid in shape and migrating to sites of inflammation. There, they secrete proteins such as cytokines, chemokines, and reactive oxygen species, which can impact synaptic plasticity and contribute to learning and memory dysfunction. These dysfunctions are associated with various conditions, including aging, AD, traumatic brain injury, HIV-related neurocognitive impairment, and psychiatric disorders such as autism, depression, and post-traumatic stress disorder (Elmore et al., 2018; Krukowski et al., 2018).

Aberrant activation of microglia and the subsequent neuroinflammation have been identified as key mechanisms underlying cognitive deficits associated with normal aging and various diseases. This suggests that microglia serve not only as immune and neural support cells in the brain but also play a crucial role in regulating synaptic structure, memory strength, and precision. Therefore, targeting microglia holds potential as a therapeutic strategy for treating cognitive disorders associated with aging, AD, and other related conditions (Lee et al., 2018; Zuckerman et al., 2018; Li et al., 2021).

### 7.3 Therapeutic targets and strategies targeting microglia

As our understanding of microglial function and metabolism deepens, several emerging trends are shaping future research in this field. One significant area of focus is the development of therapeutic strategies aimed at modulating microglial activation states. Given that microglia can exhibit both pro-inflammatory and anti-inflammatory phenotypes depending on the context, future research is likely to explore ways to fine-tune microglial responses to promote neuroprotection and reduce neuroinflammation. For instance, interventions that selectively inhibit the pro-inflammatory mTOR-HIF-1 pathway while enhancing anti-inflammatory signaling could be a promising avenue for treating neurodegenerative diseases such as AD and Parkinson’s disease (Lawson et al., 1990; Mittelbronn et al., 2001; Lalo et al., 2021).

Another promising trend is the exploration of microglial metabolism as a therapeutic target. Since microglial function is closely tied to their metabolic state, strategies that modulate glucose uptake, glycolysis, or oxidative phosphorylation could profoundly impact microglial activity and, by extension, brain health. For example, enhancing oxidative phosphorylation in microglia might promote a more quiescent, neuroprotective state, while targeted inhibition of glycolysis could reduce harmful inflammation (Barros et al., 2009; Yellen, 2018; Wu et al., 2024). Understanding the metabolic signatures associated with different microglial activation states could lead to the development of novel therapeutics that specifically target these metabolic pathways (Bélanger et al., 2011; Sun et al., 2022).

The interplay between microglial function and systemic metabolic states is gaining increasing attention. For example, dietary polyphenols (such as anthocyanins and ellagitannins in berries) can activate the AMPK/mTOR signaling pathway to inhibit NF-κB-mediated secretion of pro-inflammatory cytokines like IL-1β and TNF-*α* in microglia, thereby improving the neuroinflammatory microenvironment associated with metabolic disorders such as diabetes. Exercise or metabolic interventions may enhance CSF1R-dependent microglial ramification, promoting the release of brain-derived neurotrophic factor (BDNF) to maintain synaptic plasticity and mitigate cognitive decline linked to aging or neurodegenerative diseases (McDougall and Stewart, 2005).

### 7.4 Therapeutic potential of microglial regeneration

Furthermore, microglial regeneration as a therapeutic approach holds significant promise, with adult brain-resident nestin-positive progenitor cells capable of rapidly proliferating and differentiating into ramified microglia under CSF1R signaling. These cells secrete neurotrophic factors like brain-derived neurotrophic factor (BDNF) to promote synaptic remodeling, addressing synaptic dysfunction in neurodegenerative diseases. Advances in stem cell biology and regenerative medicine enable the generation of functional microglia from embryonic or induced pluripotent stem cells, specifically restoring dysregulated microglial networks in conditions such as Alzheimer’s disease and traumatic brain injury. This process reinstates CX3CR1-mediated synaptic pruning and complement-dependent synapse elimination, offering a novel strategy to rebuild neural circuits and cognitive function (Elmore et al., 2014).

### 7.5 Future directions: metabolic regulation and precision therapies

While microglia are well-established as central to brain immunity, recent findings reveal their mechanistic roles in synaptic plasticity via C1q/C3 complement-mediated pruning and TREM2-DAP12-dependent phagocytosis, directly regulating neuronal survival and circuit homeostasis. Future research will focus on decoding metabolic regulation—such as CSF1R-mediated resting-state energy metabolism and AMPK/mTOR-controlled activated-state metabolic remodeling—and targeting BDNF neurotrophic or TNF-α pro-inflammatory signaling to develop precision therapies for neurodegenerative diseases, leveraging microglial metabolic-immunological crosstalk (Salter and Stevens, 2017).

## 8 Emerging technologies and computational approaches in brain metabolism research

While our understanding of brain cell metabolism has been historically driven by biochemical assays and bulk tissue analysis, recent technological revolutions are enabling a more precise, dynamic, and holistic view of metabolic processes at unprecedented spatial and temporal resolutions. The integration of these advanced technologies is poised to fundamentally reshape our understanding of metabolic interactions in the brain.

Table 3 summarizes key emerging technologies and their applications, advantages, and limitations in brain metabolism research.

**Table 3:** Emerging technologies and computational approaches in brain metabolism research.

Technology	Characteristic	Application example	Advantages	Limitation
spatial omics	Transcriptome and metabolome analysis retaining spatial information (such as MALDI-IMS, DESI-MS) (Guyon et al., 2022; Jimenez-Blasco et al., 2024; Wang et al., 2025)	Map the distribution of metabolic enzymes and metabolites in brain regions such as the hippocampus and substantia nigra (Mallach et al., 2024; Bocchi et al., 2025)	Reveal cellular Regional metabolic heterogeneity	Complex data analysis Single-cell resolution Introduction of selection bias or approximation error Limited sample size
Gene-encoded biosensor	Real-time monitoring of metabolites based on fluorescent proteins (such as iATPSnFR2, GRAB sensor) (Kalinichenko et al., 2023; Deepa et al., 2024; Marvin et al., 2024)	Dynamic monitoring of ATP and glucose in living cells, imaging of neuropeptide release in specific circuits (Wang H. et al., 2023; Dong et al., 2024)	Cell specificity *In vivo* real-time observation	Low photon flux Limited applicability to modern optical profiles
Brain organoids and microfluidic models	iPSC-derived 3D organoids; microfluidic chip simulation of cell interactions and environmental control (Thomson et al., 1998; Takahashi et al., 2007; Kadoshima et al., 2013; Jo et al., 2016; Driehuis and Clevers, 2017; Fischer et al., 2019; Qian et al., 2019; Mariappan et al., 2021; Mayhew and Singhania, 2023; Mishra et al., 2024)	The interaction between brain development and pathology Dynamic simulation of neuronal metabolism after injury (Guttikonda et al., 2021; Huang and Sheng, 2022; Tan et al., 2023; Muwanigwa et al., 2024; Schmidt et al., 2025)	Humanized model Reproducible disease phenotype Controllable environment	The interpretation of dynamics under the high energy demand of human neurons is limited Other important microenvironment factors such as oxidative stress are not considered
Computational modeling and artificial intelligence	Constrained metabolic model, machine learning integration of multi-omics data (Patsatzis et al., 2025)	Neuron-astrocyte metabolic flux simulation Disease metabolic biomarker identification (Deng et al., 2024; Luo et al., 2025; Wilson et al., 2025)	System-level understanding; Predicting key metabolic nodes Integrating big data	Model-dependent assumptions

The future of brain metabolism research lies in the synergistic combination of these technologies. For example, data from spatial metabolomics can be used to parameterize computational models, and predictions from these models can then be tested using biosensors in organoids. This iterative, multidisciplinary cycle will allow us to move from descriptive observations to predictive, mechanistic models of how metabolic interactions support brain health and contribute to disease. Ultimately, this integrated approach will accelerate the discovery of novel, targeted therapeutic strategies for a range of neurological disorders.

## 9 Controversies in astrocyte-neuron metabolic coupling: the lactate shuttle and alternative viewpoints

A significant and ongoing debate in brain energy metabolism centers on the quantitative importance and primary pathway of fuel delivery to activated neurons. The ANLS hypothesis proposes a coherent model where glutamate uptake by astrocytes stimulates their glycolysis, leading to lactate production and export, which is then taken up and oxidatively metabolized by neurons to meet activity-dependent energy demands (Brooks, 2018; Dwivedi et al., 2020). This model is supported by evidence from various experimental systems, including the ability of lactate to sustain synaptic function *in vitro* (Schurr et al., 1988), the cellular localization of relevant transporters and enzymes, and *in vivo* studies using microsensors that report rapid lactate consumption coupled to oxygen utilization in the tissue parenchyma during activation (Hu and Wilson, 1997).

However, an influential alternative perspective, often termed the “non-oxidative glucose consumption” or “aerobic glycolysis” model, challenges the centrality of intercellular lactate shuttling. This viewpoint originated from observations using PET and later BOLD-fMRI, which frequently detect a larger increase in local CMRglc compared to the increase in CMRO₂ during neural activation (Fox et al., 1988). This mismatch has been interpreted to mean that a significant portion of the glucose taken up during activation is metabolized glycolytically to lactate without immediate full oxidation, and that this lactate is primarily released from the tissue (Dienel, 2012, 2017). From this perspective, neurons may satisfy their increased energy needs directly through accelerated glucose uptake and oxidative phosphorylation, with astrocyte-derived lactate playing a minimal role as an oxidative fuel under normal physiological conditions. This has led to the formulation of hypotheses such as the “efficiency tradeoff hypothesis,” which posits that the brain prioritizes the speed of ATP production via glycolysis over its efficiency during activation (Theriault et al., 2023).

The divergence between these models can be largely attributed to methodological and interpretive differences focusing on distinct physiological compartments. The ANLS hypothesis draws heavily on data from techniques that measure metabolites and oxygen directly in the brain parenchyma (e.g., microsensors, microdialysis), emphasizing the rapid dynamics of a local lactate and oxygen pool (Hu and Wilson, 1997; Almeida et al., 2004). In contrast, the non-oxidative consumption model often relies on neuroimaging techniques that infer oxygen consumption from changes in deoxyhemoglobin within blood vessels. Critics of the latter approach argue that it may fail to account for oxygen consumption from sources outside the vasculature, thus potentially overstating the “non-oxidative” nature of the glucose metabolized (Hu and Wilson, 1997; Schurr, 2018; Schurr and Passarella, 2022). This critique is substantiated by recent work highlighting the brain’s capacity for extravascular oxygen storage. For instance, the myelin sheath, constituting a large volume of the brain, has been shown to possess high oxygen solubility and can function as a capacitor-like reservoir, dampening fluctuations in oxygen availability to axons (Morelli and Scholkmann, 2023; Vervust et al., 2025). The existence of such a reservoir provides a plausible mechanism for how oxidative metabolism could proceed briskly in the parenchyma without being immediately reflected in vascular oxygen measurements (Schurr, 2025). Furthermore, the term “aerobic glycolysis” itself is a point of contention, with some arguing it perpetuates a conceptual dichotomy (aerobic vs. anaerobic glycolysis) that may not reflect the continuous nature of glycolysis ending in lactate, irrespective of oxygen availability (Schurr, 2014, 2024).

Key review articles encapsulate the skepticism towards the ANLS hypothesis, arguing that the stoichiometric requirements, kinetics of lactate transport, and in vivo metabolic data do not support a major role for lactate shuttling in meeting neuronal energy demands during activation (Dienel, 2012, 2017, 2019). A recent consensus roadmap for the field also highlights this unresolved debate (Rae et al., 2024). Conversely, comprehensive critiques argue that the evidence for aerobic glycolysis as the primary support for activation is based on a misinterpretation of BOLD signals and that the ANLS model better integrates a wider array of biochemical and physiological data (Schurr, 2014, 2018; Schurr and Passarella, 2022; Schurr, 2024).

Ultimately, resolving this controversy requires research that integrates multiple complementary techniques—high-resolution spatial metabolomics, genetically encoded biosensors in specific cell types, and advanced in vivo imaging—to simultaneously quantify metabolic fluxes, compartmentalization, and cellular interactions with high temporal and spatial precision. Incorporating the concept of significant parenchymal oxygen storage, as suggested by recent biophysical and biochemical studies (Morelli and Scholkmann, 2023; Schurr, 2025; Vervust et al., 2025), is essential for a balanced understanding of brain energetics. Acknowledging this debate is essential for a balanced understanding of brain energetics and for guiding the future research needed to elucidate the precise metabolic interactions that sustain brain function.

## 10 Outlook

### 10.1 Future directions in brain energy metabolism research

As we advance our understanding of brain energy metabolism, the emerging research trends and unsolved scientific questions reveal several promising avenues for future exploration. The intricate balance between energy supply and demand in neurons, astrocytes, and microglia, and their dynamic interactions under both normal and pathological conditions, underscores the complexity of maintaining brain health. A critical immediate step is to design experiments that can directly resolve the longstanding controversy regarding fuel utilization during brain activation. This requires moving beyond inferences from vascular signals and towards techniques capable of distinguishing metabolic fluxes within specific cellular compartments. Ideal experiments would simultaneously quantify, with high spatiotemporal resolution, key parameters such as: (1) lactate and glucose dynamics in astrocytes and neurons using genetically encoded biosensors; (2) oxygen concentration and consumption within the tissue parenchyma itself, not solely in the vasculature; and (3) the contribution of local oxygen reservoirs, such as those in myelin sheaths (Morelli and Scholkmann, 2023; Vervust et al., 2025), to meeting transient metabolic demands. By directly testing whether activated neurons primarily oxidize astrocyte-derived lactate versus other substrates, and by accurately accounting for all sources of oxygen (vascular and extravascular), such approaches could provide definitive evidence to reconcile the conflicting models (Tietze, 1969; Bolaños et al., 2010). Moving forward, the integration of cutting-edge technologies such as multi-omics approaches, advanced imaging techniques, and computational modeling will be pivotal in unraveling these complexities.

### 10.2 Decoding metabolic heterogeneity for precision targeting

While single-cell technologies have revealed metabolic diversity in neurons and glia, most studies remain static. The dynamic metabolic reprogramming (e.g., rapid glycogen mobilization in astrocytes during ischemia) and its role in disease progression are poorly understood. For instance, astrocytic LDHA downregulation in Alzheimer’s disease may not only impair energy supply but also directly suppress synaptic plasticity genes via epigenetic modifications (e.g., H3K18 lactylation)(Yao et al., 2023). Future studies should develop time-resolved metabolic tracing technologies to uncover how metabolic heterogeneity drives degeneration in specific neuronal subtypes.

### 10.3 Mechanistic conflicts and translational challenges in metabolic interventions

Although ketogenic diets show promise in animal models, clinical outcomes are inconsistent. Recent work found that β-hydroxybutyrate (BHB) enhances mitochondrial function but may exacerbate neuroinflammation by inhibiting histone deacetylases (Sun et al., 2022). Such dual effects highlight the need to stratify patients by disease stage and metabolic status. Moreover, brain-region specificity of metabolic interventions (e.g., hypothalamic sensitivity to ketones vs. cortical reliance on lactate) remains unexplored, limiting broad application. Integrated platforms (e.g., brain organoids coupled with clinical cohorts) are needed to define therapeutic windows and contraindications.

### 10.4 Spatiotemporal dynamics of metabolic-immune crosstalk

Microglial metabolic plasticity (e.g., M1 phenotype relying on glycolysis vs. M2 phenotype preferring oxidative phosphorylation) is well-documented, but its regulation of synaptic pruning remains unclear. For example, in Parkinson’s disease, does TREM2-mediated *α*-synuclein phagocytosis by microglia depend on lactate levels? Live imaging reveals lactate-induced rapid microglial morphological changes, yet the signaling pathways (e.g., via monocarboxylate transporters or GPCRs) are unresolved (Salter and Stevens, 2017; Dilmore et al., 2023). Targeting these metabolic-immune interfaces may yield dual-action therapeutics with anti-inflammatory and neuroprotective properties.

### 10.5 From systemic metabolism to personalized medicine

The gut-brain axis links microbial short-chain fatty acids (SCFAs) to astrocytic glutathione metabolism, but less than 1% of SCFAs reach the brain due to blood–brain barrier heterogeneity (Sharon et al., 2016). Personalized approaches must address two conflicts: (1) discrepancies between systemic biomarkers (e.g., plasma BHB) and intracerebral metabolic states; (2) non-linear associations between genotypes and metabolic phenotypes. Promising strategies include nanoparticle-mediated targeted delivery and CRISPR activation of endogenous metabolic enzymes, combined with AI-driven prediction of individual metabolic responses.

### 10.6 Personalized medicine and neurodegeneration

As the field moves towards personalized medicine, understanding individual variability in brain metabolism will be crucial for developing targeted treatments. There are sex-specific differences in brain energy metabolism. Genetic and environmental factors can influence metabolic responses and disease susceptibility. By identifying patient-specific metabolic profiles, researchers can design more effective, personalized therapeutic approaches that address the unique metabolic challenges faced by individuals with neurodegenerative diseases.

### 10.7 Novel metabolic interactions among neurons, astrocytes, and microglia

#### 10.7.1 Neuron: metabolon at the synapse

Beyond relying on global somatic energy production, emerging evidence suggests the existence of localized “metabolons” within neurons, particularly at presynaptic terminals (Li and Sheng, 2022). We propose that these terminals may possess independently regulated glycolytic enzymes and mitochondrial networks, allowing them to generate ATP rapidly in response to high-frequency firing without solely depending on axonal transport from the cell body. This synaptic metabolic autonomy could be crucial for sustaining vesicle cycling and calcium homeostasis during intense cognitive tasks. Investigating how this local metabolic machinery is regulated, potentially by activity-induced calcium signals, may reveal why synapses exhibit varying vulnerabilities to energy stress in neurodegenerative diseases.

#### 10.7.2 Astrocyte: systemic metabolic sensing

Astrocytes may function as master sensors of the body’s systemic energy status within the brain. Beyond their well-known response to local neuronal activity, we hypothesize that circulating hormones like ghrelin (hunger signal) and leptin (satiety signal) directly bind to receptors on hypothalamic astrocytes. This binding could modulate astrocytic glycogen storage and lactate production, thereby translating peripheral metabolic information into changes in local neural circuit activity and influencing feeding behavior. This positions astrocytes as critical integrators in the gut-brain axis, orchestrating whole-body energy balance by adjusting brain energy supply accordingly.

#### 10.7.3 Microglia: the metabolic checkpoint

The decision of microglia to either support or prune neurons and synapses may be guided by a “metabolic checkpoint.” We postulate that microglia constantly assess the metabolic fitness of neighboring neurons by sensing specific metabolites released from dysfunctional cells. For instance, abnormal accumulation of succinate or specific lipids from stressed neurons could act as “eat-me” signals, triggering phagocytic activity in microglia. This mechanism would ensure the removal of energetically incompetent neurons, maintaining circuit health. Dysregulation of this metabolic checkpoint could lead to either excessive synaptic loss or the survival of damaged neurons, contributing to neurodevelopmental or neurodegenerative pathologies.

#### 10.7.4 Integrated perspective: the metabolic triad

**Figure 4:**
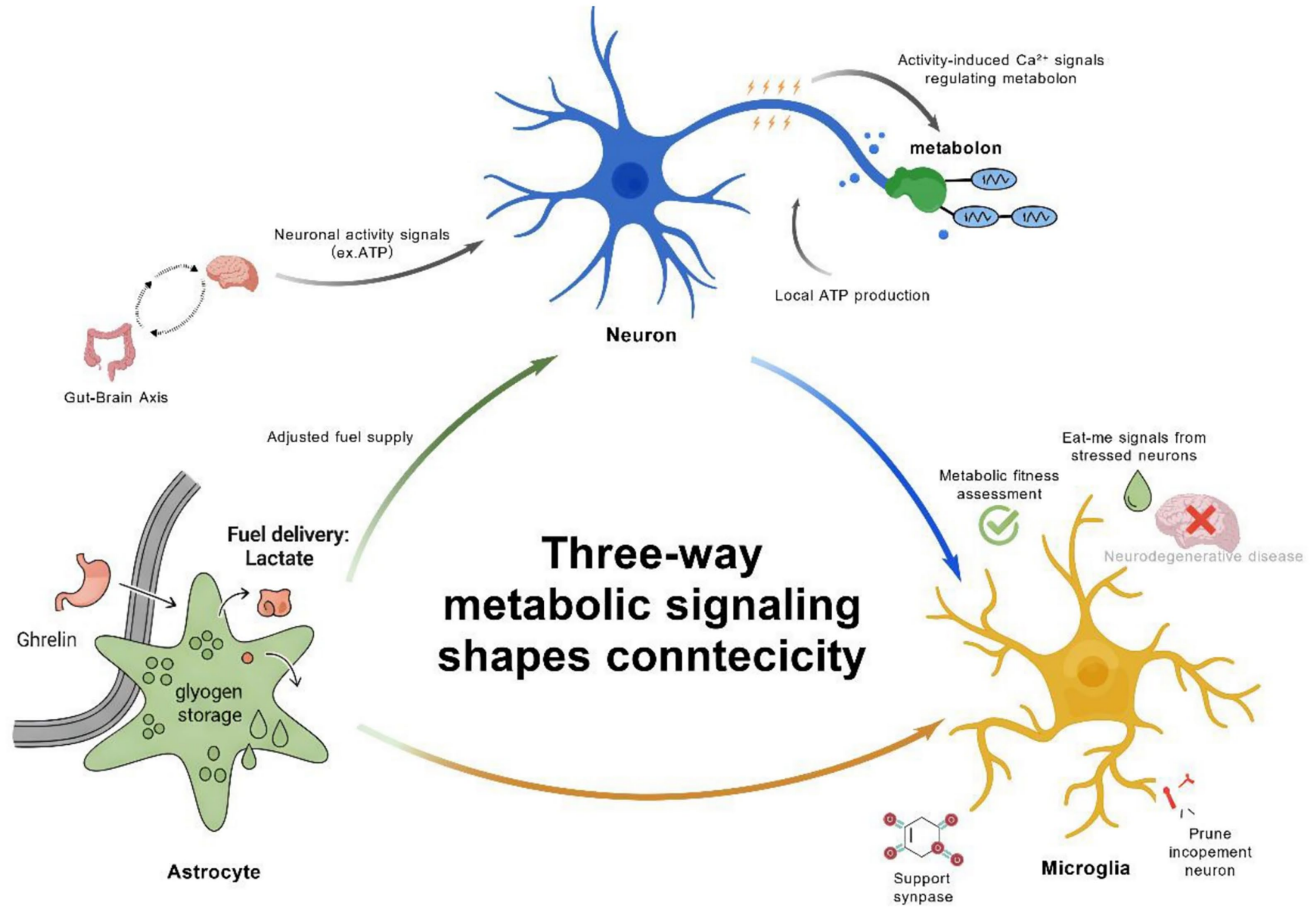
The metabolic triad: three-way metabolic signaling shapes neural connectivity. This integrated model illustrates the dynamic metabolic crosstalk between neurons, astrocytes, and microglia in regulating synaptic health, energy supply, and immune surveillance (by Biogdp).

These concepts culminate in a novel “metabolic triad” model (Figure 4). In this framework, neuronal activity initiates the dialogue by releasing signals (e.g., ATP). Astrocytes respond by adjusting fuel (lactate) delivery, while microglia, guided by their metabolic checkpoint, monitor the interaction and decide to either support the synapse or prune it based on its metabolic health. This dynamic, three-way communication highlights that brain energy metabolism is not merely about fuel delivery but is an integral signaling language that shapes neural connectivity and function.

## 11 Discussion

### 11.1 Energy metabolism in neurons

Progress in neuronal energy metabolism research has been substantial, yet several critical constraints impede further translation. It is debated whether and to what extent neurons depend on astrocytic lactate, given evidence of their autonomous glycolytic capacity in specific contexts (Tang, 2018; Díaz-García and Yellen, 2019). A major constraint is the predominant use of reduced preparations (e.g., *in vitro* models, acute slices), which fail to capture the integrated milieu of the intact brain. Additionally, metabolic heterogeneity among neuronal subtypes is widely recognized but difficult to resolve with current spatially-limited techniques. Ultimately, extrapolating findings from animal models of neurodegeneration to humans is complicated by differences in disease chronology and etiology.

### 11.2 Energy metabolism of astrocytes

The study of astrocyte metabolism is complicated by dynamic functional states and translational gaps. Their substantial regional heterogeneity challenges the concept of a uniform astrocytic phenotype, with direct implications for metabolic support. In neurodegenerative contexts, a critical dichotomy emerges: a loss of support function contrasts with reactive, potentially inflammatory metabolic reprogramming. The molecular triggers and progression of this shift are poorly resolved. These therapeutic ambitions are further tempered by the practical difficulties of achieving cell-type-specific modulation without disrupting homeostasis or compromising delivery across the blood–brain barrier.

### 11.3 Relationship between astrocyte and neuron energy metabolism (including lactate shuttle and glutathione shuttle)

While models of astrocyte-neuron metabolic coupling, such as the lactate and glutathione (GSH) shuttles, are well-supported, their universality and pathophysiological relevance remain subjects of debate. For example, the quantitative contribution of the lactate shuttle likely varies by brain region and neural activity, and the interplay between its roles as an energy substrate and a signaling molecule (e.g., in epigenetic regulation) is poorly defined. Technological limitations pose a major barrier: real-time, quantitative *in vivo* tracking of metabolite flux is hindered by trade-offs between spatiotemporal resolution, specificity, and invasiveness in current methodologies. Regarding the GSH shuttle, despite its central role in antioxidant defense, clinical trials aiming to boost brain GSH levels through precursor supplementation have shown inconsistent outcomes. This is attributable to the blood–brain barrier, variable cellular uptake, and the irreversibility of oxidative damage in late disease stages. Collectively, these gaps highlight the need for next-generation tools capable of precise metabolic mapping and for validation in more human-relevant models, such as brain organoids.

### 11.4 Microglia

The primary obstacle in microglial metabolism research is its profound plasticity and context-dependency. The classic M1/M2 paradigm is insufficient to describe their phenotypic continuum, making corresponding metabolic states highly dynamic and difficult to target predictably. Compounding this is the poorly understood metabolic crosstalk within the microglia–neuron-astrocyte triad, where signaling mechanisms and functional outcomes are unclear. This functional ambiguity extends to disease, where causal relationships between metabolic reprogramming and pathology are hard to dissect. Translational efforts face an additional barrier: while CSF1R inhibitors allow experimental microglial manipulation, recreating their physiological complexity—including precise spatial distribution and functional heterogeneity—presents a significant challenge for therapeutic development.

## 12 Conclusion

The future of brain energy metabolism research lies in addressing these unsolved questions through a multidisciplinary approach that combines advanced technologies with a deep understanding of cellular and systemic metabolism. By exploring the regulatory networks that sustain neuronal and glial function, and by developing targeted metabolic interventions, we can make significant strides in preventing and treating neurodegenerative diseases, ultimately improving brain health and cognitive function across the lifespan.

## Glossary

2DG-IR: 2-deoxyglucose infrared (glucose analog)
4D-FastDIA: 4-dimensional fast data-independent acquisition
Aβ: Amyloid-beta
AD: Alzheimer’s disease
ALS: Amyotrophic Lateral Sclerosis
AMPK: AMP-activated protein kinase
ANLS: Astrocyte-neuron lactate shuttle
APC/C-Cdh1: Anaphase-promoting complex/cyclosome and its coactivator Cdh1
ATP: Adenosine triphosphate
BDNF: Brain-Derived Neurotrophic Factor
BHB: β-hydroxybutyrate
BK channel: Large conductance Ca^2+^ activated potassium channel
CAP: Compound action potential
CNS: Central Nervous System
CREB: cAMP response element-binding protein
CSF1R: Colony-stimulating factor 1 receptor
CX3CR1: CX3C chemokine receptor 1
EAAC1: Excitatory amino acid carrier 1 (neuronal glutamate transporter)
EAAT1/2: Excitatory amino acid transporter 1/2 (astrocytic glutamate transporters)
fAHP: Fast afterhyperpolarization
FDG: 2-fluoro-2-deoxy-D-glucose
G6P: Glucose-6-phosphate
GABA: Gamma-aminobutyric acid
GCL: Glutamate cysteine ligase
Glc-6-P: Glucose-6-phosphate
GLT-1/GLAST: Na^+^-dependent transporters
GLUT: Glucose transporter (e.g., GLUT1, GLUT3, GLUT4)
GPCR: G protein-coupled receptor
GRAB: GPCR Activation-Based (biosensor)
GSH: Glutathione
GSK3β: Glycogen synthase kinase 3 beta
HIF-1*α*: Hypoxia-inducible factor 1-alpha
HK2: Hexokinase 2
iPSC: Induced pluripotent stem cell
LDH: Lactate dehydrogenase (e.g., LDHA, LDHB)
LTD: Long-term depression
LTP: Long-term potentiation
MCT: Monocarboxylate transporter (e.g., MCT1, MCT2, MCT4)
MCU: Mitochondrial calcium uniporter
mLDH/cLDH: Mitochondrial/cytosolic lactate dehydrogenase
MPTP: 1-methyl-4-phenyl-1,2,3,6-tetrahydropyridine (neurotoxin)
mTOR: Mechanistic target of rapamycin
NAD+/NADH: Nicotinamide adenine dinucleotide (oxidized/reduced)
NADPH: Nicotinamide adenine dinucleotide phosphate (reduced)
NAC: N-acetylcysteine
NF-κB: Nuclear factor kappa-light-chain-enhancer of activated B cells
Nrf2: Nuclear factor erythroid 2-related factor 2
PD: Parkinson’s disease
PDK4: Pyruvate dehydrogenase kinase 4
PFK: Phosphofructokinase
PFKFB3: 6-phosphofructo-2-kinase/fructose-2,6-bisphosphatase 3
PGC-1α: Peroxisome proliferator-activated receptor gamma coactivator 1-alpha
PKM1/PKM2: Pyruvate kinase M1/M2 isoforms
PLC: Phospholipase C
PPP: Pentose phosphate pathway
ROS: Reactive Oxygen Species
SCFAs: Short-chain fatty acids
SFO: Subfornical organ
SIRT1: Sirtuin 1
SUR1: Sulfonylurea receptor 1 (component of K-ATP channel)
Sxc^−^: Cystine/glutamate antiporter (system xc^−^)
TCA: Tricarboxylic acid
TNF-α: Tumor necrosis factor-alpha
TREM2: Triggering receptor expressed on myeloid cells 2
